# Shear‐Responsive Red Blood Cell Carriers for Targeted Drug Delivery to Constricted Arteries

**DOI:** 10.1002/smll.74818

**Published:** 2026-07-31

**Authors:** Danielle Nemcovsky Amar, Rukiye Tuna, Tirosh Mekler, Merav Belenkovich, Dmitry Korneyev, Yevgeniy Kreinin, Anat Glozman, Josué Sznitman, Donald E. Ingber, Zixiang Leonardo Liu, Netanel Korin

**Affiliations:** ^1^ Department of Biomedical Engineering Technion Israel Institute of Technology Technion City Haifa Israel; ^2^ Department of Chemical & Biomedical Engineering FAMU‐FSU College of Engineering Tallahassee Florida USA; ^3^ Wyss Institute for Biologically Inspired Engineering at Harvard University Boston Massachusetts USA; ^4^ Vascular Biology Program Department of Surgery Boston Children's Hospital and Harvard Medical School Boston Massachusetts USA; ^5^ Harvard John A. Paulsson School of Engineering and Applied Sciences Cambridge Massachusetts USA; ^6^ Fischell Department of Bioengineering University of Maryland College Park Maryland USA

**Keywords:** cardiovascular disease, cell mechanics, drug delivery, hemodynamics, mechano‐therapeutics, red blood cells, stenosis

## Abstract

Red blood cells (RBCs) are widely utilized as natural drug delivery systems due to their unique mechanical properties, which enable prolonged circulation and physiological function. This study examines a biophysical strategy that employs shear‐responsive RBC (sr‐RBC) carriers to mechanically release drugs at stenotic arteries. Intracellularly loaded sr‐RBCs exhibited shear‐dependent cargo release when perfused through stenotic arterial models, while no release was observed in unconstricted vessels. Notably, the sheared sr‐RBCs retained their membrane integrity and CD47 expression while maintaining their ability to traverse microvascular networks without release when perfused through a microfluidic model simulating pulmonary capillaries. Multiscale modelling of RBC suspension fluid dynamics demonstrates a strong positive correlation between the sr‐RBC drug release and the overall RBC membrane stretching induced by the high shear rates at various stenotic percentages. Sr‐RBCs and mechanosensitive cell carriers may enable new avenues for treating life‐threatening arterial constrictions and other vascular diseases.

## Graphical abstract

Red blood cells are harnessed as mechanoresponsive carriers that exploit the elevated shear stress of stenotic arteries to transiently open membrane pores and release encapsulated therapeutics selectively at sites of arterial narrowing. By coupling vascular hemodynamics with RBC membrane mechanics, a hemodynamically activated platform for localized vascular drug delivery is established.

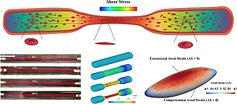

## 1 Introduction

Red blood cells (RBCs) have been widely investigated as drug carriers because of their abundance, biocompatibility, and long circulation time in the bloodstream. Designed by nature to efficiently transport oxygen throughout the body, RBCs provide an ideal, non‐immunogenic vehicle for therapeutic delivery. Two primary strategies have been developed to harness RBCs for drug delivery: surface attachment and intracellular loading [1, 2, 3, 4]. Using surface attachment, for example, RBCs loaded with thrombolytic drugs such as tissue plasminogen activator (tPA) have been explored as a prophylactic modality for preventing clot formation [5, 6]. Recently, cellular hitchhiking, where nanoparticles are attached to the surface of RBCs, has shown highly promising results for first‐pass delivery to target organs. For example, uptake in the lungs increased by 120‐fold, and studies have demonstrated potential applications in lung and brain‐targeted delivery [7]. Other work inspired by the unique characteristics of RBCs developed discoidal polymeric nano‐constructs mimicking erythrocytes that enhanced the delivery and efficacy of tPA by 90% at a clinical dose [8]. Another approach involves loading therapeutic agents directly into RBCs, either in their free form or as particles. Autologous RBC intracellular drug encapsulation technologies have been developed to load RBCs with various therapeutic agents for a wide range of pathologies [9, 10]. Several such therapeutics, including specific formulations, have reached clinical trials, such as L‐asparaginase‐loaded RBCs for treatment of pancreatic cancer, and dexamethasone‐loaded RBCs for treatment of inflammatory bowel disease [11]. Generally, encapsulating therapeutic agents inside RBCs provides a shield against immune system recognition and facilitates the delivery of hydrophobic drugs. Regardless of the drug‐loading method, preserving the native mechanical flexibility of RBCs is essential to ensure prolonged circulation, minimal immune response, and efficient therapeutic delivery [12, 13].

RBCs are highly deformable, which allows them to withstand a wide range of shear stresses and flow through narrow capillaries [8, 14, 15] and splenic slits [16, 17]. However, for human RBCs, at pathological shear stresses around 150 dyne/cm^2^, transient nanoscale pores can form on their surface. At shear stress levels exceeding 450 dyne/cm^2^, a hemolysis threshold is reached, where the membrane ruptures irreversibly [18, 19]. Recently, transient pore formation due to membrane stretching while flowing in constricted microchannels has been exploited as a mechanism to load therapeutic agents into RBCs and other cell types [20]. However, so far, mechanical disruption of cells, and RBCs in particular, has not been explored as a strategy to trigger drug release at sites of disease. Interestingly, previous studies investigating the release of adenosine triphosphate (ATP) from native RBCs in response to shear stress demonstrated a direct correlation between high shear stress and increased ATP levels in in vitro microfluidic models [21, 22]. Hence, RBC carriers could potentially be designed to act as a mechanoresponsive drug delivery platform, where shear stress serves as a trigger for controlled drug release.

Several cardiovascular disease conditions, including stenotic arteries, are associated with pathological elevated shear stresses (>100 dyne/cm^2^), which go well beyond any normal shear stress [23, 24]. These elevated shear stresses may lead to pathological events, including shear‐induced Von Willebrand factor activation that causes high‐shear thrombosis [25] and shear‐induced RBC membrane rupture that causes RBC hemolysis [19, 26]. Leveraging this biophysical pathological feature, shear drug delivery systems have been proposed that aim to deliver drugs to stenotic arteries based on the high shear at these sites. For example, shear‐activated nanoparticle aggregates, 3–4 µm in diameter, that disperse to their single nanosized particles when exposed to high shear stress, such as in stenotic vessels, have been developed [27]. A clot‐busting formulation of the shear‐activated nanoparticle aggregates was shown to be highly effective for thrombolytic therapy in several in vivo disease models [28]. Other approaches have been studied, which include shear‐responsive nanocarriers releasing antiplatelet medication [29, 30].

Despite these advances, mechanosensitive RBC‐based release has never been demonstrated. Furthermore, no prior study has linked transient RBC membrane area‐strain dynamics under pathological shear to controlled intracellular drug release. To this end, we present sr‐RBC carriers that release their cargo at stenotic arteries via transient pore opening in their membrane, induced by the hemodynamic forces at these sites. We demonstrate, via in vitro physiological flow experiments, selective shear‐triggered release in stenotic arterial models, while showing minimal release under normal physiological flows such as those in capillary network models. Moreover, combining experimental shear‐triggered RBC drug‐release data with multiscale membrane‐dynamics simulations, we find that release closely correlates with the substantial RBC deformation seen in stenotic flow, supporting our strategy for localized delivery to arterial narrowings.

## 2 Results and Discussion

### 2.1 Shear‐Triggered Release From RBC Flowing in Constricted Arteries‐Biophysical Principle

**FIGURE 1:**
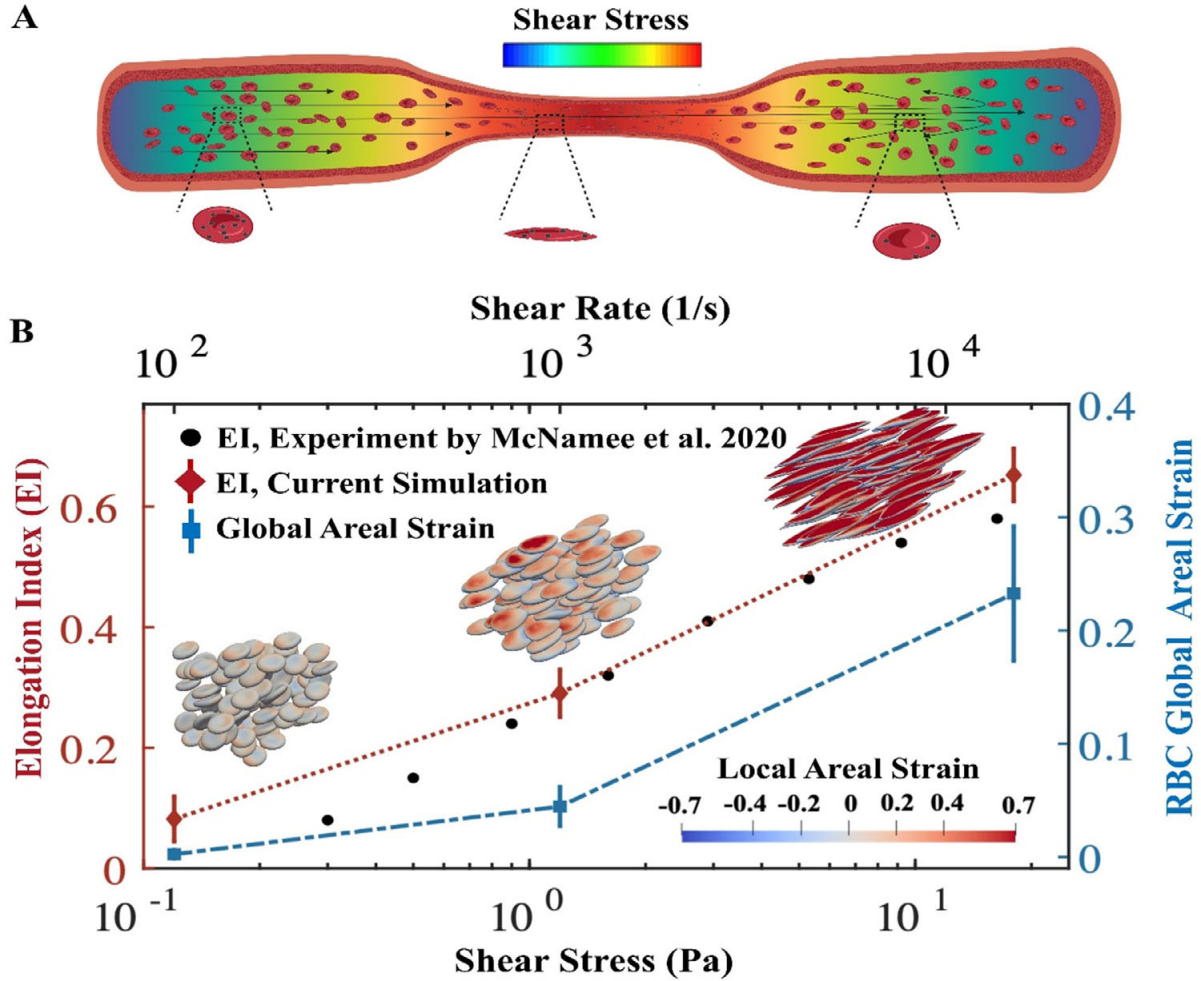
The biophysical principles of the shear‐responsive RBC (sr‐RBC) carriers for localized drug delivery to stenotic arteries. (A) Schematic illustration showing sr‐RBC drug carriers traveling through a stenotic artery and experiencing pathologically elevated shear stresses. Under such elevated shear conditions, RBCs deform substantially, which can lead to pore formation in their membranes and induce local drug release at the stenosis sites. (B) Simulation of sr‐RBC suspensions at 20% haematocrit under normal and pathological levels of simple shear flows. With physiological shear (<1000 1/s), RBCs maintain their biconcave shape. As shear rate increases beyond the physiological level (corresponding to the stenotic region), RBCs elongate and local membrane stretches to form pores. RBC deformation quantified using the elongation index shows good agreement with experimental measurements obtained via laser diffractometry (ektacytometry) reported by McNamee et al. [31]. The inset shows the configuration of the RBC suspensions under different shear levels. The color of the RBC membrane shows the local areal strain level. The global area strain is quantified as the integral of the local area strain for each RBC.

The operating principle of sr‐RBC carriers relies on the mechanical deformation experienced by RBCs as they traverse narrowed arteries of the circulation, as shown in Figure 1A [18, 19]. Under normal flow conditions, the wall shear rate (WSR) remains below 1000 s^−^^1^, and RBCs retain their characteristic biconcave morphology, exhibiting mild elongation with increasing shear. As sr‐RBCs pass through a constricted artery, they experience pathologically elevated shear rates that can be an order of magnitude higher than those in normal circulation. As shown in Figure 1B, 3D multiscale simulations of RBC suspensions show significant deformation occurring under pathological shear levels, where RBC deformation is determined by the elongation index (EI) calculated by EI  =  (L  −  W)/(L  +  W), where L represents the length of the major (longer) axis of the cell and W is the minor (shorter) axis of the cell. The EI of RBCs increases to values of 0.08 and 0.29, respectively, ultimately reaching 0.65 at a shear rate of 15 000 s^−^^1^, in good agreement with experimental ektacytometry data reported by McNamee et al. [31]. Although RBCs possess an excess surface area, over 40% more than a sphere of the same volume, which allows them to deform significantly under physiological conditions without membrane area changes. Extreme shear forces can induce significant alterations in membrane area, see inset RBC suspension (20% hematocrit) snapshots in Figure 1B showing the local areal strain (AS) defined as AS  = (A_i_ − A_o_)/A_o_ , in which the A_i_ and A_o_ are the current and initial area of a local RBC membrane patch, respectively. Once a critical shear threshold is exceeded, pathological deformation leads to membrane area changes that, above a certain criterion, can lead to transient pore formation. While EI has been utilized as a solid metric to quantify RBC elongation, RBC AS offers a direct measurement of the threshold for RBC membrane pore formation. These biomechanical processes highlight the shear‐sensitive nature of sr‐RBCs and support their potential as mechanically activated drug carriers for vascular diseases involving stenotic regions.

### 2.2 Shear‐Triggered Release of sr‐RBCs Flowing Through a Stenotic Artery and sr‐RBC Membrane Integrity

**FIGURE 2:**
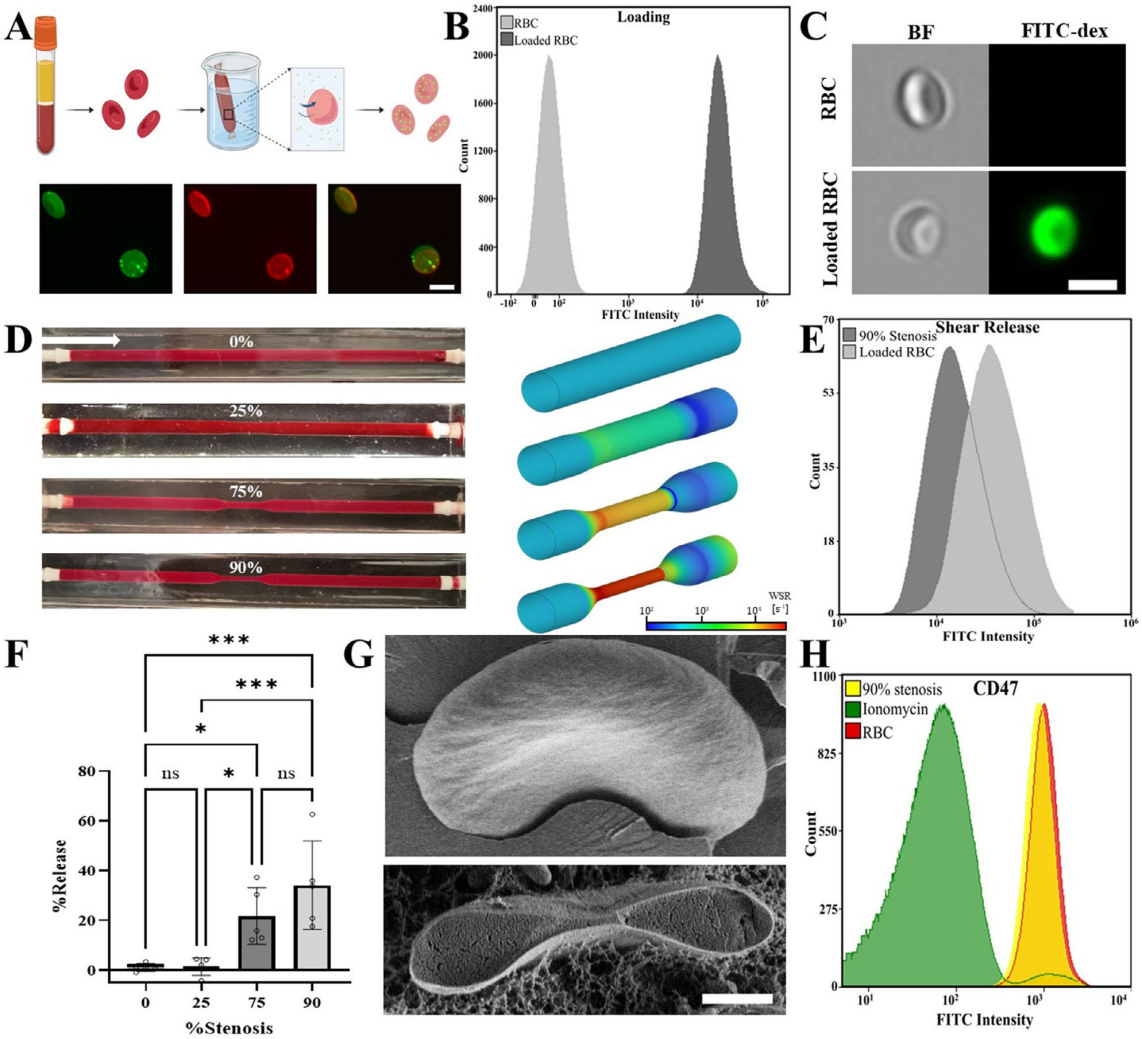
Shear‐triggered release from sr‐RBC flowing through a stenotic artery model. (A) Top: illustration of the sr‐RBC drug loading procedure via hypotonic swelling. Bottom: Fluorescence confocal images of sr‐RBC loaded with FITC‐Dex (20 kDa, in green) and stained with VybrantTM Dil, for membrane labelling (in red). Scale bar 5 µm. (B) Histogram of the FACS measurement of the FITC intensity in loaded and unloaded RBCs. Loaded RBCs display high levels of FITC, indicating successful encapsulation of FITC‐Dex. (C) Representative ImageStream brightfield and fluorescent images of RBC and loaded sr‐RBC. Scale bar 10 µm. (D) Photo of real‐sized arterial models recapitulating straight vessels (0 stenosis), mild stenotic (25% stenosis), moderate stenotic (75% stenosis), and severe stenotic (90% stenosis), from top to bottom, perfused with blood and sr‐RBCs at physiological flow conditions and their corresponding CFD wall shear rate, to the right. As expected, with a higher degree of stenosis, WSR and velocity increase, and recirculatory zones appear in the post‐stenotic region. (E) Histogram of FACS of encapsulated RBCs' pre‐ and post‐perfusion in a 90% stenosis model. Following perfusion through the stenotic model, there is a decrease in fluorescent intensity, indicating a release of FITC‐Dex from the sr‐RBC; the arrow represents a shift in FITC intensity. (F) Percentage of released FITC‐Dex from sr‐RBC post‐perfusion in real‐sized stenotic models. Significant release of FITC‐Dex is noticed when sr‐RBC flows through stenotic models compared to the straight model. Statistical significance was determined by one‐way ANOVA, ^*^: *p* < 0.05, ^**^: *p*‐value< 0.01 (*n* = 5 biological replicates). (G) CryoSEM images of RBC (top) and loaded sr‐RBC (bottom). Sr‐RBC kept the biconcave shape after the loading process. Scale bar 2 µm. (H) Histogram of FACS quantification of CD47 expression on RBC membranes. RBCs and 90% stenosis show positive expression of CD47, whilst RBCs treated with Ionomycin are negative for CD47, suggesting that RBCs preserve CD47 expression and membrane integrity.

To investigate hemodynamic effects on sr‐RBCs, in vitro experiments were conducted using transparent models replicating arterial constrictions. Sr‐RBC carriers encapsulating fluorescein isothiocyanate‐Dextran (FITC‐Dex) (MW 20 kDa) were produced via osmotic loading followed by membrane resealing (see Methods and Figure 2A and Figure S1). Fluorescence confocal imaging and flow cytometry analysis confirmed effective drug loading, with sr‐RBCs displaying strong green fluorescence compared to unloaded samples (Figure 2B,C). To further characterize sr‐RBC loading performance, we quantified the encapsulation efficiency (%EE) of FITC‐Dex cargoes (see Supporting Information). The %EE was 12.8% ± 9.1% for 20 kDa FITC‐Dex and 4.4% ± 3.8% for 70 kDa FITC‐Dex, indicating successful encapsulation of both molecular weight cargos. In addition, cargo retention was evaluated under static storage conditions over 72 h. Sr‐RBCs retained 66% ± 8% and 62% ± 18% of the initially loaded 20 and 70 kDa FITC‐Dex, respectively (Figure S2). Most cargo loss occurred during the first 24 h, during which fluorescence decreased by approximately 35% ± 5%. Following this initial phase, cargo retention remained relatively stable for the remainder of the observation period. Fluorescent membrane labeling demonstrated that RBC morphology was preserved (Figure 2A bottom). To assess shear‐induced drug release, sr‐RBCs were perfused through physiologically relevant arterial models with varying stenosis levels (See Figure 2D and Supporting Information and Figure S3). Computational fluid dynamics (CFD) simulations confirmed increased shear forces within stenotic regions, with higher velocity gradients and recirculatory zones appearing downstream of severe stenoses. Maximum WSR values were approximately 1500, 16 000, and 70 000 s^−^^1^ for 25%, 75%, and 90% stenosis, respectively (Figure 2D). Flow cytometry measurements post‐perfusion revealed a significant decrease in FITC fluorescence in RBCs that traversed a 90% stenotic model, indicating shear‐induced drug release (Figure 2E). Quantitative analysis showed that cargo release increased sharply in the 75% and 90% stenotic models, while remaining low in the 25% stenosis and straight vessel conditions (20% ± 10% for 75% and 34% ± 15% for 90% stenosis over 1 h of perfusion, *p*< 0.05 and *p* < 0.01, respectively), see Figure 2F. Additionally, Cryo‐SEM images showed that sr‐RBCs retained their normal biconcave morphology (Figure 2G and Figure S1C). To ensure that dialysis and perfusion experiments did not alter CD47 levels, a key RBC membrane protein that acts as a self‐recognition signal preventing immune clearance, flow cytometry analysis was performed on RBCs treated with ionomycin, a calcium ionophore that induces eryptosis, RBCs, and sr‐RBCs post‐perfusion through a 90% stenotic model. Positive FITC signals confirmed that CD47 remained intact post‐shearing, unlike sr‐RBCs treated with ionomycin, which lacked CD47 expression (Figure 2H). To further investigate sr‐RBC ability to evade immune clearance, a phagocytosis assay was performed. THP‐1 cells were differentiated using PMA and incubated with native RBCs, sr‐RBCs, and RBCs treated with ionomycin (Supporting Information and Figure S4). Macrophages incubated with sr‐RBCs exhibited phagocytosis levels comparable to those observed with native RBCs, whereas cells incubated with ionomycin‐treated RBCs showed significantly increased phagocytosis (Figure S4b and Movie S5). Additionally, supporting characterization indicated that the sr‐RBC loading process preserved membrane surface charge and did not induce cell aggregation, while resulting in a modest reduction in mean corpuscular volume (Figure S5). These findings establish sr‐RBCs as effective mechanosensitive drug carriers capable of targeted release under pathological shear conditions. Additional measurements of sr‐RBC over 72 h demonstrate that sr‐RBC retained their morphological features (Figure S6) and MCV had no significant decrease, as seen in Figure S7a. Moreover, there was no significant decrease in CD47 expression after 24 h (Figure S7b,c).

### 2.3 sr‐RBCs Demonstrate Physiological Navigability and Cargo Retention in a Microfluidic Capillary Network Mimicking the Pulmonary Micro‐Circulation

**FIGURE 3:**
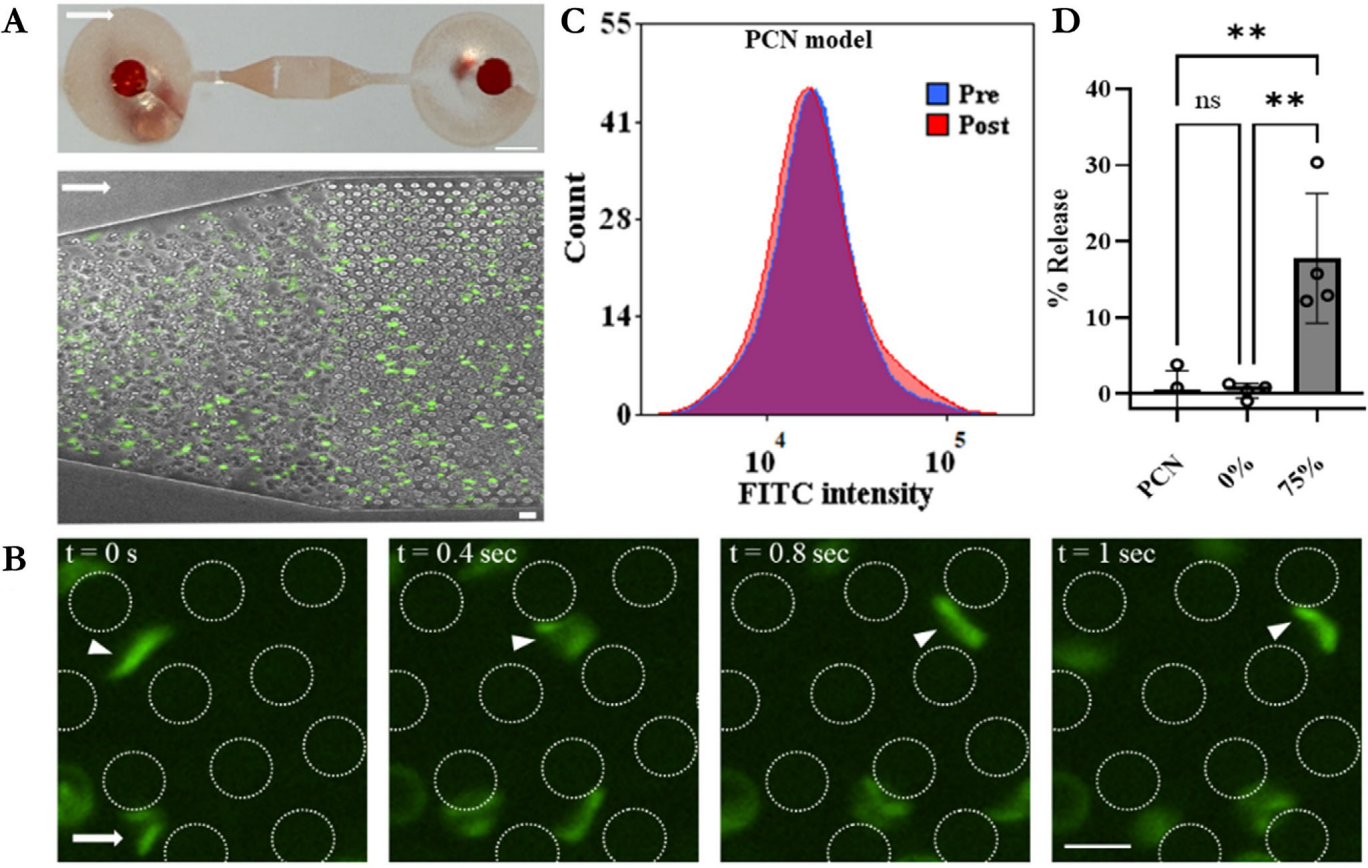
Sr‐RBC navigating through a Pulmonary Capillary Network (PCN) microfluidic model. (A) Front view of the PCN model with RBC recapitulating pulmonary capillary networks containing 5 µm diameter pillars with 5 µm spacings. Scale bar 450 µm, on the bottom superimposed close‐up of brightfield and fluorescent (FITC) microscopy images of a PCN model perfused with 30% haematocrit composed of ten percent FITC‐Dex‐loaded sr‐RBCs mixed with unloaded RBCs. The arrow represents the flow direction. Scale bar 20 µm. (B) Time‐lapse fluorescent microscopy images of loaded sr‐RBC (white arrowhead) navigating through the PCN model (circles indicate PCN pillars) under physiological flow, demonstrating their deformability capabilities. The arrow represents the flow direction. Scale bar 5 µm. (C) Histogram of flow FACS of the FITC intensity in loaded sr‐RBC, before and after perfusion in the PCN model. FITC levels stayed consistent, suggesting minimal to no release of FITC from sr‐RBC. (D) Quantification of FITC‐Dex released in the PCN model vs. a real‐sized straight model and a 75% stenosis model. In both the PCN and no stenosis conditions, RBC released was not significant as opposed to when sr‐RBC traveled through a stenotic model. Statistical significance was determined by one‐way ANOVA, ^*^: *p* < 0.05, ^**^: *p*‐value< 0.01 (*n* = 4 biological replicates).

To verify that sr‐RBCs retain their navigability and that shear‐triggered release of FITC‐Dex was specifically induced by high shear in stenotic arteries, sr‐RBCs were perfused through a microfluidic model of the pulmonary capillary network [32] (PCN) (see Figure 3A top and Supporting Information and Figure S8). Sr‐RBCs were mixed with unloaded RBCs to establish physiological hematocrit levels and perfused through the PCN model (Figure 3A, bottom). Time‐lapse imaging demonstrated that sr‐RBCs navigated the PCN model while maintaining physiological deformability (Figure 3B and Movie S1). Flow cytometry analysis of sr‐RBCs collected post‐perfusion confirmed retention of fluorescence intensity, indicating that perfusion through capillaries did not induce FITC‐Dex release (Figure 3C). Quantitative comparison with straight real‐sized models showed no significant difference, suggesting that sr‐RBC shear‐triggered release occurs primarily in stenotic arteries under pathological shear conditions (Figure 3D). These results indicate that sr‐RBCs remain stable under physiological microvascular flow and release their cargo primarily under pathological shear conditions.

### 2.4 Global RBC Areal Strain at the Stenosis Correlates With the RBC‐Encapsulated Drug Release

**FIGURE 4:**
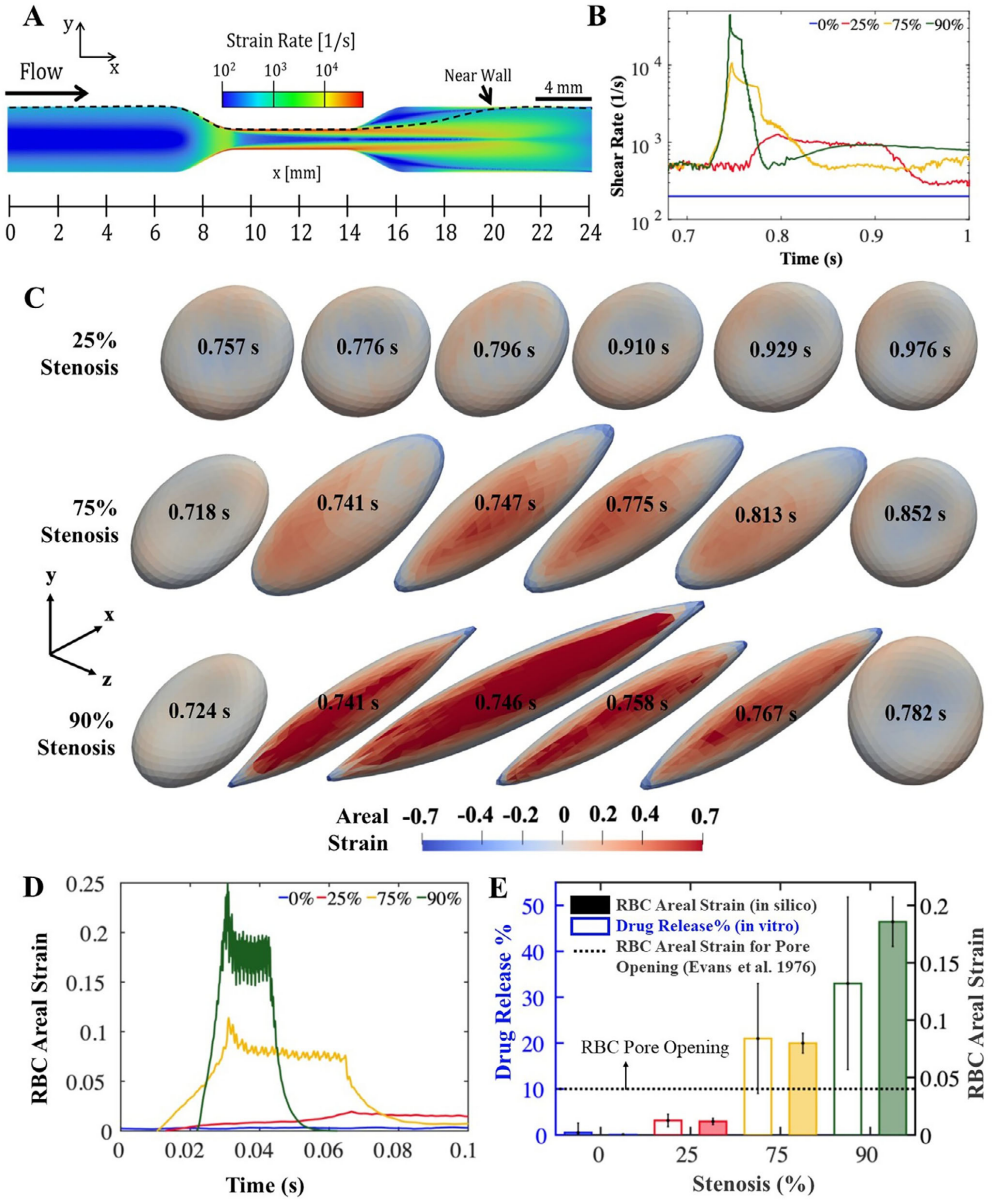
Global RBC areal strain at the stenotic region correlates with the RBC‐encapsulated drug release through stenosis. (A) Shear rate distribution in the 90% stenosis model central plane and the path‐line for the representative RBC near the wall that is of interest in the study. (B) The shear rate history experienced by the near‐wall traversal RBC through stenotic arteries of different stenosis percentages (0, 25%, 75%, and 90%). (C) Cellular blood flow simulation shows the transient RBC deformation and local areal strain distribution on the cell membrane when traveling through stenoses of different percentages (25%, 75% and 90%). (D) Time histories of the total RBC membrane areal strain for RBCs flowing through different stenoses. (E) Total RBC areal strains sampled (solid bars) from the stenotic region for various stenosis degrees correlate with the experimental drug release% (empty bars). The dotted line denotes the upper threshold for RBC membrane areal strain (0.04% or 4%) for pore opening measured experimentally by Evans et al. 1976 [33].

To better understand the kinetics and deformation characteristics associated with sr‐RBC shear‐triggered release, we performed multiscale simulations of transient sr‐RBC suspension dynamics (Methods section, Supporting Information & summary of RBC mechanical properties used in Table S1). First, CFD simulations mapped radial shear rate variations within the constricted models, and the radial velocity gradient of RBC carriers near the vessel wall (i.e., 10 µm from the wall at the unconstricted entrance area) was extracted under different stenosis conditions. Figure 4A presents the strain rate distribution at the central plane of the 90% stenosis model, showing a two‐order‐of‐magnitude increase in strain rate at the constricted region on a logarithmic scale. As shown in Figure 4B, near‐wall sr‐RBCs experienced a 6‐, 53‐, and 225‐fold increase in radial velocity gradient when passing through 25%, 75%, and 90% constrictions, respectively. Additionally, an RBC path line demonstrates flow separation near the vessel wall. Cellular blood flow simulation shows the transient RBC deformation kinetics and local areal strain (AS) distribution on the cell membrane as the sr‐RBC travels through different stenosis conditions (Figure 4C). To quantify sr‐RBC membrane deformation under these dynamic conditions, we tracked local changes in the membrane AS using mesoscale simulation. The simulations revealed extensive transient sr‐RBC deformation within stenotic arteries, with the RBC radius increasing to 9.1 µm (127.5%) and 12.6 µm (215%) for 75% and 90% stenosis, compared to its baseline 4 µm in unconstricted conditions and the 25% stenosis results which showed slight increase of 4.4 µm (10%) (Figure 4C and Movies S2–S4). Time‐resolved simulations show that the globally averaged extensional areal strain (AS) increases with stenosis severity, with instantaneous peaks of 6% (±4%), 7% (±6%), 27% (±17%), and 47% (±32%) for 0%, 25%, 75%, and 90% stenosis, respectively (Figure 4D). Although pore formation is a local membrane event, the global areal strain can provide a measure of whether transient local membrane stretching exceeds experimentally established pore‐opening thresholds. Interestingly, experimental drug release correlates well with the extensional Global AS averaged along the stenotic throat region at various stenosis degrees, as shown in Figure 4E, where the empty bars are experimental drug release (%) and the solid bars are quantified extensional AS (%) from coarse‐grained RBC simulations. From 0% to 90% stenosis, the drug release % (empty bars) is 0.5%, 3.2%, 21%, and 33%, respectively, while the corresponding total RBC areal strain (solid bars) is 0.0002, 0.012, 0.08, and 0.19, respectively. The dotted line denotes the upper threshold for RBC membrane areal strain (0.04% or 4%) for pore opening measured experimentally by Evans et al. 1976 [33].

## 3 Discussion

This study describes an approach in which RBCs are used as drug carriers whose release behavior is governed by hemodynamic conditions. This study investigates the encapsulation and release profile of FITC‐Dex from human RBC carriers in response to elevated WSR. We demonstrate significant release of FITC‐Dex from sr‐RBCs after perfusion through mild and severe stenotic models. In contrast, sr‐RBCs perfused through the PCN microfluidic model did not release their cargo, suggesting that sr‐RBC membrane response is distinct when flowing through pathological stenotic arteries compared to under physiological conditions, including within the smallest capillaries of the body through which the entire cardiac output circulates. In straight, non‐stenotic arteries, wall shear stress (WSS) increases approximately proportionally with flow rate, whereas its dependence on vessel size is much stronger (τ_*w*_∝*Q*/*R* ^3^ under laminar assumptions). Thus, while exercise increases cardiac output, blood velocity, and wall shear stress in healthy arteries, the increase in WSS generally remains within the physiological range (10–70 dyn/cm^2^). In contrast, arterial stenosis can increase WSS by 10–100 fold, over a short length, imposing severe deformation on RBCs. Furthermore, clinical studies demonstrate that increased cardiac output elevates laminar WSS, which triggers vascular remodeling and improves endothelial function, whereas high WSS associated with stenosis can cause undesired bio‐damages and effects such as platelet activation [34, 35, 36]. It is also worth noting that the shear rates generated in the 90% stenosis model are not representative of healthy arterial flow but are consistent with reported values in severe arterial narrowing. In contrast to exercise‐induced increases in flow, which elevate shear stress only several‐fold, drastic lumen reduction in high‐grade stenosis can lead to order‐of‐magnitude increases in local shear rate. Accordingly, the 90% stenosis model is intended as a worst‐case pathological scenario to explore deformation‐activated release thresholds. Lower‐grade stenoses produce more moderate shear levels, consistent with the reduced release observed experimentally [37].

The release kinetics of sr‐RBCs are directly linked to the mechanical deformation induced by pathological shear stress levels. Our in vitro perfusion studies revealed a shear‐dependent release profile, where FITC‐Dex release increased with the severity of stenosis. Complementary multiscale simulations demonstrate that the release kinetics of sr‐RBCs are directly linked to the mechanical deformation induced by pathological shear stress levels. For the stenotic flow conditions considered in our study, the RBC transit time at the stenotic throat is above 20 ms. As shown by Razizadeh et al. [26], the pore formation time scale is on the order of 10 ms, which is significantly shorter than the RBC transit time. Therefore, it should still be acceptable to treat the RBC overall stretching when flowing through the high shear throat section as quasi‐static in terms of the pore formation It is worth noting that although the AS values observed for RBCs flowing through a stenosis exceed those reported under steady‐state conditions, prior studies have shown that pore‐formation kinetics differ substantially between quasistatic loading [33], and high‐rate deformation [19, 26, 38, 39, 40, 41]. In particular, the critical areal strain for pore opening depends not only on the strain magnitude but also on the rate at which the strain changes [26]. This aspect in sr‐RBCs should be further explored in detail in future more mechanistic studies.

Hemodynamic‐based drug delivery was previously introduced by Korin et al., who engineered shear‐responsive nanoparticle aggregates that break apart under high shear and release drug‐carrying nanoparticles in stenotic sites [27]. Our study builds on this concept but uniquely leverages RBCs as natural drug carriers. Using RBCs instead of synthetic carriers presents several advantages, including their intrinsic deformability, prolonged circulation time, abundance, and potential for autologous administration, which minimizes immune responses. Moreover, previous studies demonstrated that engineered RBCs could preserve biophysical properties and physiological function. Li et al. showed that engineered RBCs as gas carriers still maintained their key properties (circulatory, membrane integrity, etc.) [42]. Consistent with the preservation of these biological properties, macrophage uptake experiments performed using PMA‐differentiated THP‐1 cells demonstrated that sr‐RBCs exhibited phagocytosis levels comparable to native RBCs, while ionomycin‐treated RBCs showed significantly elevated uptake. These findings suggest that the loading process does not substantially alter membrane‐associated properties involved in immune recognition and clearance. Moreover, other approaches utilizing RBCs as drug carriers affected by mechanical stimuli include hitchhiking RBCs [43], where nanoparticles are attached to RBC surfaces. However, while highly effective, this method primarily targets capillary networks downstream of the injection site through a single‐pass targeting approach, making it unsuitable for delivering therapeutics to large arteries or serving as a circulating prophylactic system that triggers release upon arterial constriction.

A wide range of therapeutics could benefit from localized, hemodynamically triggered release using sr‐RBCs. Drugs whose efficacy depends on achieving high local concentrations, such as thrombolytics (e.g., tPA), vasorelaxant agents for treating vasospasm, anti‐inflammatory biologics acting on activated endothelium, and anti‐platelet or anti‐thrombotic drugs, could be delivered more safely by restricting their release to pathological high‐shear regions [44, 45]. Targeted delivery in this manner has the potential to enhance therapeutic potency while minimizing systemic exposure and off‐target effects. In addition, RBC‐based carriers offer practical translational advantages: they can be prepared autologously, reducing immunogenicity, and they are compatible with established clinical RBC encapsulation technologies that have already reached human trials. These features position sr‐RBCs as a clinically accessible mechanotherapeutic platform for diseases involving pathological flow disturbances. The translational potential of red blood cell–based drug carriers is supported by prior clinical progress using autologous RBCs for controlled drug release [46]. Extension of the present approach to in vivo studies will require adaptation to animal models, as species‐dependent differences in RBC membrane mechanics and circulation stability are expected to influence deformation thresholds and carrier durability [47]. Future work will therefore focus on characterizing these mechanical differences, optimizing loading protocols, and evaluating release behavior under pathological flow conditions in relevant animal models.

Our experimental setup demonstrates the feasibility of sr‐RBCs as shear‐responsive carriers; however, the precise mechanistic pathways governing their release require further investigation. Moreover, our current in vitro models are simplified and lack the full complexity of the vasculature and its components. Future studies will need to validate our findings in more complex models, including large animal studies, to assess circulation dynamics and clearance mechanisms. Additionally, our study focused on the loading and release profile of 20 kDa FITC‐Dex. FITC‐Dex (20 kDa) was chosen as an initial model cargo due to its widespread use in RBC encapsulation studies and its representative size relative to many peptide and protein therapeutics. The additional demonstration of shear‐activated release for 70 kDa FITC‐Dex indicates that deformation‐mediated release is not limited to a single molecular weight, although release efficiency decreases with increasing cargo size (Figure S9). This trend is consistent with established studies showing that cargo diffusivity and steric constraints influence both retention and release from RBC carriers. In contrast, small molecules (<10 kDa) are known to exhibit passive leakage from RBCs even in the absence of mechanical activation, limiting their suitability for deformation‐triggered delivery strategies [48, 49]. While the presented study demonstrated the capability of mechanoresponsive release of large molecules from sr‐RBCs, future studies should further investigate the release kinetics of smaller molecular weight therapeutics. Additionally, the release of specific drugs should be examined individually as their physicochemical properties can affect the interaction with the RBC and the shear release profile. The encapsulation efficiency data further highlight the need to optimize the loading process and emphasize the influence of cargo properties on loading performance. Furthermore, retention studies demonstrated that most of the cargo loss happens in the first 24 h after fabrication. This study focused on the immediate application after fabrication; future studies should further optimize storage conditions and solutions to improve long‐term retention and to advance toward clinical translation.

The intracellular concentration of encapsulated cargo can be tuned to a certain extent by adjusting the initial mass during hypotonic loading; however, loading efficiency is ultimately constrained by RBC volume and membrane integrity. Together, these results highlight both the versatility and the practical limits of RBC‐based carriers and motivate future optimization tailored to specific therapeutic classes [50, 51, 52]. Future work should investigate therapeutic agents with different molecular weights and their release kinetics in response to shear stress. While substantial FITC‐Dex release was observed under high shear, the large standard deviation across donors highlights the heterogeneity of human blood. Improving clinical reproducibility will likely require additional RBC isolation or standardization steps to reduce inter‐donor variability. Furthermore, although sr‐RBCs retained their shape and CD47 levels post‐perfusion, additional markers such as phosphatidylserine‐ an indicator of apoptosis‐ should be evaluated to fully assess cell stability and longevity in circulation.

Collectively, the results suggest that sr‐RBCs can serve as mechanically activated carriers for localized drug release in high‐shear vascular environments. Looking ahead, this approach could be expanded to target various vascular diseases characterized by altered hemodynamics, including vasospasms post‐aneurysm rupture, atherosclerosis, and cardiovascular devices such as left ventricular assist devices (LVADs), cardiopulmonary bypass (CPB), and extracorporeal membrane oxygenation (ECMO). By integrating advanced biomaterials, engineering strategies, and computational modelling, future iterations of sr‐RBC carriers could be designed with enhanced loading capacities, tuneable release profiles, and precision targeting capabilities. Ultimately, mechanoresponsive drug delivery strategies like sr‐RBCs hold the potential to transform the treatment of cardiovascular diseases by enabling site‐specific therapy with minimal off‐target effects.

## 4 Experimental Section/Methods

### 4.1 Blood Preparation

Ethical approval was obtained for the experiments with blood samples from human subjects (Rambam Medical Center Institutional Review Board (IRB) RMB‐0413‐21). For blood experiments, whole blood was taken from healthy human volunteers (consent from all participants was obtained prior to the research), and supplied by the Israeli National Blood Service (Rambam Medical Center Institutional Review Board (IRB) RMB‐0413‐21). Red blood cells were separated from other blood components using a centrifuge at 700 g for 10 min. RBC were resuspended in 10 mL of phosphate‐buffered saline without calcium and magnesium (PBS‐, Sigma). FITC‐Dex solution was prepared by mixing fluorescein isothiocyanate‐Dextran (FITC‐Dex, Sigma) with PBS to a final concentration of 4 mg/mL. Both solutions were mixed and injected into a dialysis bag (70 K cutoff). For the lysis step, the dialysis bag was placed in a container filled with hypotonic solution containing 10% PBS with double distilled water (DDW) and incubated for 2 h at 4°C while stirring. Afterward, for the resealing process, the dialysis bag was transferred to isotonic solution containing PBS with calcium and magnesium (PBS+, Sigma) and allowed to stir overnight at room temperature. Finally, the RBC mixture was washed at 700 g for 10 min to attain FITC‐loaded RBCs.

### 4.2 Real‐Sized Stenotic Models and Experimental Setup

To study the mechano‐responsiveness of RBCs in physiological and pathophysiological conditions, transparent, real‐sized 3D arterial models were fabricated. Models were first designed by computer‐aided design (CAD) software. Four models were designed: a straight model, a 25% stenosis model, a 75% stenosis model, and a 90% stenosis model, each 11 cm in length with a 4 mm diameter in the straight part. The 25% stenosis model has a 3.46 mm diameter, the 75% stenosis model has a 2 mm diameter at the stenotic region, and the 90% stenosis model has a 1.26 mm diameter at the stenotic region, based on area stenosis percentage. 3D mold was printed from 3DM‐ABS resin using an Elegoo‐Mars 3D printer; resin residue was removed, and the model's surface was smoothed and polished. Then, a mixture of polydimethylsiloxane (PDMS, Sylgard 184, silicone elastomer) was degassed and allowed to cure overnight at room temperature. Afterward, the models were placed in acetone to dissolve the resin mold. The finished models were placed in 65°C for at least 48 h to ensure complete acetone evaporation. For further details, see Supporting Information. 3D models were connected to a closed‐circuit perfusion system (Watson Marlow 530C, Watson Marlow, UK) that mimics arterial flow as previously described (Supporting Information and Figure S10) [53, 54]. To ensure a constant flow profile, a damper (250 mL bottle with a ThermoFischer Scientific Nalgene 3‐port filling/venting cap) was connected after the peristaltic pump to dampen the peristaltic pulse. Five microliters of loaded RBCs were mixed with PBS (‐/‐); the solution was perfused for 1 h, and samples were taken pre‐ and post‐perfusion to measure the percentage of FITC‐Dex released from the RBCs.

### 4.3 Macroscopic Hemodynamic Simulation

Computational fluid dynamics (CFD) simulations were conducted on four different models representing various degrees of stenosis (healthy, 25%, 75%, and 90%). The simulations were carried out using Ansys Fluent, and the mesh was generated with Fluent Meshing. For each model, polyhedral cells were created with five inflation layers at the wall. To perform a mesh convergence test, three meshes were generated for the severe stenosis case (90%), with cell counts ranging from approximately 1.4–4.2 million cells (see Supporting Information for details). To ensure that a fully developed flow reaches the stenosis region in the CFD, the length between the inlet and the start of the stenosis was extended to approximately 12 times the inlet diameter (See Figure S11 for flow profiles along the model entrance). An incompressible, Newtonian fluid with blood properties (ρ = 1060 kg/m^3^ and µ = 3.5 cP) was used in all simulations, with an inlet flow rate of 200 mL/min, zero pressure at the outlet, and no‐slip conditions on the walls. Steady‐state conditions were assumed, and the viscous k‐ω turbulence model was employed using the SIMPLE scheme to solve the numerical simulation. To obtain the spatial derivatives of the axial velocity at a specific location within the domain, massless particles were seeded near the wall and at the center, and data from their trajectories were recorded. For the 25% and 75% stenosis cases, a laminar viscosity model produced results similar to the shear stress transport (SST) k model, indicating predominantly laminar flow. In the 90% stenosis case, however, the Reynolds number reached approximately 1000 at the throat, suggesting the onset of transition and post‐stenotic flow instabilities. The SST model is therefore more appropriate for capturing such effects, as it has been shown in previous studies to accurately describe arterial blood flow [55] and also when validated using experimental data [56]. Blood was modeled as a Newtonian fluid because, under the flow conditions considered in this study, the local shear rates are sufficiently high for blood viscosity to remain effectively constant, as demonstrated from rheological measurements [57].

### 4.4 HPF Cryo‐Specimens Preparation and Imaging

The samples were fixed by high‐pressure freezing (HPF). In this technique, about 1 µL of the samples is sandwiched between two metal discs (3 mm diameter, 0.1 mm cavities). Assembled “sandwiches” were high‐pressure frozen with liquid nitrogen at about 210 MPa (Leica, EM ICE, Liechtenstein). The frozen specimens were then mounted on a specialized sample table under liquid nitrogen and transferred by a cooled cryo‐transfer shuttle under vacuum (EM VCT500; Leica) to a freeze‐fracture system (EM ACE900; Leica), where it was kept at −150°C. In the ACE chamber, the sandwiches were split open using a cooled knife, heated to −100°C for 3 min to sublime a thin water layer for morphology enhancement, and then were transferred by the precooled and pumped VCT500 shuttle to the precooled cryo‐stage of the HR‐SEM. Fractured cryo‐specimens were imaged on a Zeiss Ultra Plus high‐resolution SEM, equipped with a Schottky field‐emission gun and with a Leica VCT500 cold‐stage maintained at −150°C. Specimens were imaged at low acceleration voltages of 0.7–1 kV, low‐dosage of about 130pA beam current, and working distances of 3–5 mm. We used both the Everhart Thornley (“SE2”) and the in‐column (“InLens”) secondary electron imaging detectors. Low‐dose imaging was applied to all specimens to minimize radiation damage.

### 4.5 Flow Cytometry

For flow cytometry analysis, loaded RBCs were isolated from the perfusion mixture after perfusion. As described above, RBCs were loaded with FITC‐Dex, followed by perfusion in different stenosis models recapitulating pathological shear stress. As a negative control, unloaded RBCs were used. Measurements were conducted using a flow cytometer (BD, LSR Fortessa, Becton Dickinson, USA) equipped with a 488 nm excitation laser. To ensure data integrity, cell doublets were excluded via forward scatter width (FSC‐H) vs. forward scatter area (FSC‐A) analysis, and dead cells were identified and removed based on their diminished size and low granularity in the side scatter area (SSC‐A) vs. FSC‐A plot. Flow cytometry data analysis was performed in FCS Express (DeNovo). Additionally, to ensure that reported values reflect intracellular cargo release from intact carriers rather than artifacts, flow cytometry analysis was gated to exclude cellular debris and aggregates, resulting in the exclusion of approximately 2% of the population. For further details, see Supporting Information and Figure S12.

### 4.6 RBC Characterization CD47

To ensure our procedure has little effect on RBC membranes, CD47 levels were measured. Briefly, CD47 is a glycoprotein located in many of the cell's membranes and has a critical role in immune system self‐recognition [58, 59, 60]. Basal levels of CD47 ensure that cells are recognized as native cells. Loaded RBCs were incubated in the dark with 10 µg/mL of anti‐CD47 for 30 min at room temperature. Then, the cells were washed, and the samples were taken for FACS analysis within 15 min. For the positive control, we used ionomycin to induce erythropoiesis. We can see that all samples besides the Ionomycin test kept the same level of CD47.

### 4.7 Zeta Potential

RBC and sr‐RBC membrane potential: both RBC were resuspended in Hanks' Balanced Salt Solution (HBSS) (without calcium and magnesium), at 0.1% haematocrit and analyzed using Zetasizer Nano ZS (Malvern Panalytical, Malvern, UK) [61].

### 4.8 MCV

Mean corpuscular volume was obtained by Mindray BC‐30 Vet (Mindray Animal Care, China) [62, 63].

### 4.9 PCN Fabrication and Experimental Setup

To establish that FITC‐Dex release from the RBCs is a result of changes in shear stress, a microfluidic model of PCN that was recently developed by Stauber et al. was used [32]. This microfluidic device is based on Fung and Sobin's “shear flow” model [64], a mesh of 5 µm cylindrical pillars with 5 µm spacing between them. The fabrication of the master mold is explained in detail by Stauber et al. [32]. For further details, see Supporting Information. Then, the device was fabricated from PDMS using soft lithography. PDMS was degassed and allowed to cure; inlet and outlet holes were punched, and PDMS was sealed using O_2_ plasma. Prior to the flow experiment, PCN models were prewashed with ethanol followed by PBS and coated with 1% bovine serum albumin to prevent air bubbles and non‐specific binding. The experimental setup was detailed previously; briefly, the system comprised an inverted epi‐fluorescent microscope (Ti–Eclipse, Nikon, Japan) and a pressure‐driven system (Fluigent MFCS‐EZ, France). Sr‐RBCs were resuspended in PBS to achieve 33% haematocrit. Working flow was 4 kPa, which corresponds to physiological velocities in the pulmonary capillaries. Perfused samples were collected and taken for further FACS analysis.

### 4.10 Mesoscopic Cellular Blood Flow Simulations and Numerical Setup

A multiscale molecular and cellular blood flow solver that effectively captures the dynamics and binding of blood cells and macromolecules is employed for the current study [65, 66]. The flow field for both the blood plasma and cytoplasm is modeled using the lattice Boltzmann method [67]. The red blood cell (RBC) membrane dynamics and deformation are modeled through a coarse‐grained spring‐based viscoelastic membrane mechanics model [68, 69]. This molecular and cellular blood flow solver has been extensively validated against theory and experiments in various scenarios for cellular blood flow and clotting [65, 66, 68, 70, 71, 72, 73].

RBC simulations are conducted in a cubic computational domain of size 24^3^ µm^3^ that is unbounded and triply periodic, imposed via the Lees–Edwards boundary condition [74]. A time‐varying shear rate is imposed to drive the flow of RBCs along the x‐direction, where the transient WSR is obtained from macroscopic hemodynamic simulations. An RBC is modeled as a deformable capsule that adopts a biconcave discoidal shape under stress‐free equilibrium, with the maximum diameter being 8 µm. The membrane nonlinear mechanics capture the RBC membrane viscoelasticity with physiological RBC shear elasticity, bending stiffness, and membrane viscosity. The internal viscosity of RBC is set to five times that of plasma (1.2 cP), matching the physiological RBC conditions.

### 4.11 Statistical Analysis

All results are presented as mean and standard deviation (SD). Data were analyzed using a one‐way ANOVA or an unpaired Student's *t*‐test with unequal variance, using PRISM software, as noted in the figure legends. Statistical significance was considered when *p* < 0.05.

## Author Contributions

Tirosh Mekler: methodology, investigation, Writing – review and editing, validation, software. Merav Belenkovich: methodology, Writing – review and editing. Danielle Nemcovsky Amar: conceptualization, methodology, investigation, Writing – original draft, Writing – review and editing, data curation, validation, formal analysis, visualization. Yevgeniy Kreinin: methodology, Writing – review and editing, visualization. Josué Sznitman: methodology, Writing – original draft, Writing – review and editing. Dmitry Korneyev: methodology, Writing – review and editing. Donald E. Ingber: conceptualization, resources, funding acquisition, Writing – original draft, Writing – review and editing, supervision. Rukiye Tuna: conceptualization, methodology, investigation, Writing – review and editing, Writing – original draft, data curation, validation, formal analysis, visualization, software. Anat Glozman: methodology, Writing – review and editing. Zixiang Leonardo Liu: conceptualization, methodology, investigation, resources, funding acquisition, Writing – original draft, Writing – review and editing, project administration, supervision. Netanel Korin: conceptualization, methodology, investigation, resources, funding acquisition, Writing – original draft, Writing – review and editing, project administration, supervision.

## Funding

This project has received funding from the European Research Council (ERC) under the European Union's Horizon 2020 research and innovation programme (grant agreement No. 101002057) and the Wyss Institute for Biologically Inspired Engineering at Harvard University.

## Ethics Approval and Patient Consent Statements

Ethical Approval Was Obtained For the Experiments With Blood Samples from Human Subjects (Rambam Medical Center Institutional Review Board (IRB) RMB‐0413‐21). For blood Experiments, Whole Blood Was Taken from Healthy Human Volunteers (consent from All Participants Was Obtained Prior to the research) and Supplied By the Israeli National Blood Service (Rambam Medical Center Institutional Review Board (IRB) RMB‐0413‐21).

## Conflicts of Interest

DEI and NK are co‐founders of ShearFlow Inc. and are inventors on issued and/or pending patents related to shear‐responsive drug delivery system. All other authors declare no conflict of interest.

## Supporting information




**Supporting File 1**: smll74818‐sup‐0001‐SuppMat.pdf.

**Supporting File 2**: smll74818‐sup‐0002‐MovieS1‐S5.zip.

## Data Availability

The data that support the findings of this study are available from the corresponding author upon reasonable request.
