# Supplementary material for: Shear‐Responsive Red Blood Cell Carriers for Targeted Drug Delivery to Constricted Arteries
Source: Small. 2026 Jul 31;22(52):e74818. doi: 10.1002/smll.74818 (PMC13580257; doi:10.1002/smll.74818)
Supplement: Supplementary file 1 [file SMLL-22-e74818-s002.pdf]

Supplementary Material For:

Shear-Responsive Red Blood Cell Carriers

for Targeted Drug Delivery to Constricted Arteries

Danielle Nemcovsky Amar[1,±], Rukiye Tuna[2,±], Tirosh Mekler[1], Merav Belenkovich[1], Yevgeniy Kreinin[1], Dmitry Korneyev[1], Anat Glozman[1], Josué Sznitman[1], Donald E. Ingber[3-5], Zixiang Leonardo Liu[2*], Netanel Korin[1,6*]

[1]Department of Biomedical Engineering, Technion Israel Institute of Technology, Technion City, Haifa 3200003, Israel.

[2]Department of Chemical & Biomedical Engineering, FAMU-FSU College of Engineering, Tallahassee, Florida, 32310, USA.

[3]Wyss Institute for Biologically Inspired Engineering at Harvard University, Boston, Massachusetts, USA.

[4]Vascular Biology Program, Departments of Pathology & Surgery, Boston Children's Hospital and Harvard Medical School, Boston, Massachusetts, USA.

[5]School of Engineering and Applied Sciences, Harvard University, Cambridge, Massachusetts, USA.

[6]Fischell Department of Bioengineering, University of Maryland, College Park, MD,United States

*Corresponding author. Netanel Korin, Email: korin@bm.technion.ac.il ; Zixiang Leonardo Liu, Email: leo.liu@eng.famu.fsu.edu

Supplementary material includes:

Supplementary material text

Figure S1-12

Movie V1-5

Table S1

RBC imaging

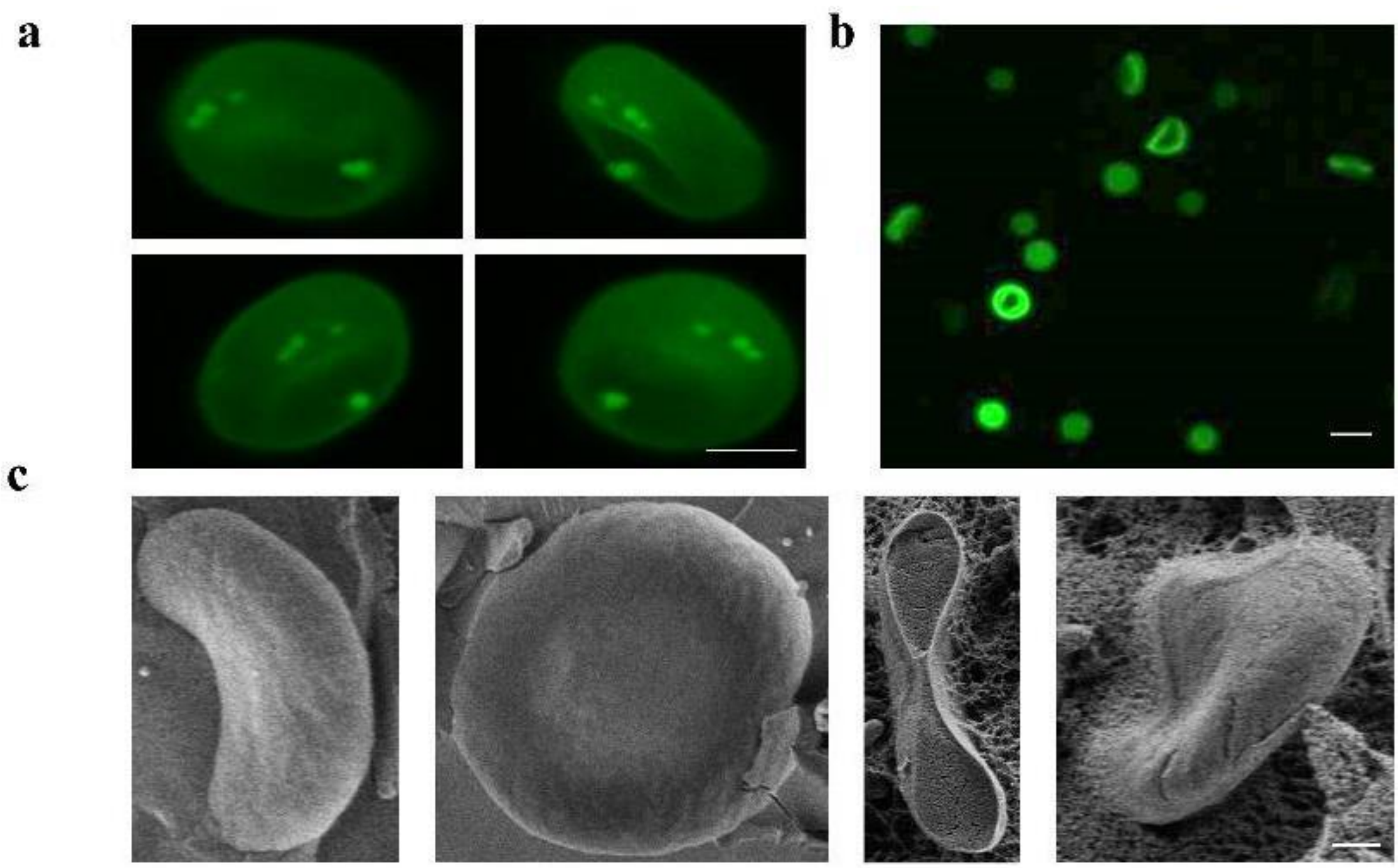

Figure S1. Representative images of RBC and sr-RBC (a) confocal microscopy–based 3D reconstructions, scale bar 2 µm (b) Fluorescence confocal images of sr-RBC loaded with FITC-Dex (20 kDa, in green), scale bar 10 µm (c) CryoSEM images of RBC (two on the left) and loaded sr-RBC (two on the right), scale bar 1 µm.

Encapsulation efficiency

Following the loading process, sr-RBCs were collected and incubated with double distilled water (DDW) to induce cellular lysis. Then solution was centrifuged to remove cellular debris, and the resulting supernatant was analyzed using plate reader (Synergy N1, BioTek®) at 490 nm. A calibration curve was prepared for each blood sample, and encapsulation efficiency (%EE) was calculated as the ratio between the encapsulated FITC-dex concentration measured from each experiment (C_{exp}) and the initial FITC-dex concentration ($C_{initial}$), as described in the following equation:

$$\%EE = \frac{C_{exp}}{C_{initial}}$$

The %EE of sr-RBCs loaded with 20 kDa and 70 kDa FITC dex was 12.8±9.1 (n = 6 biological replicates). and 4.4±3.8, (n = 3 biological replicates), respectively.

Cargo retention in static conditions

FITC-dex retention inside the sr-RBC was evaluated over a course of 72 hours, where samples were measured every 24 hours, Figure S2. Sr-RBC were mixed with SAGM solution containing mannitol, adenine and dextrose, in 30% hematocrits, stored at 4^0C. The fluorescent intensity of sr-RBC was measuring every 24 hours and normalized to the fluorescent intensity at 0 hours. Initial decrease in fluorescent intensity was observed during the first 24 hours for both 20 and 70 kDa, indicating passive cargo leakage, while after 24 hours the fluorescent signal was stabilized.

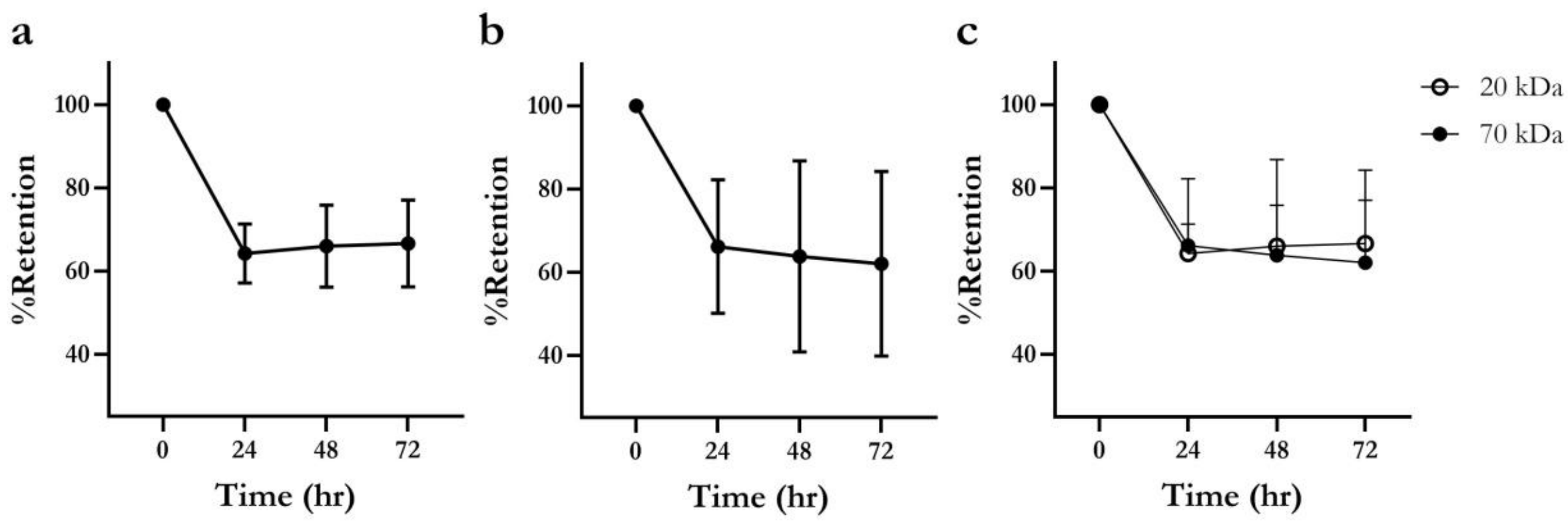

Figure S2. Cargo retention of sr-RBCs loaded with 20 and 70 kDa FITC-dex over 72 hours under static storage conditions at 4^0C. Cargo retention for (a) 20 kDa FITC-dex (b) 70 kDa FITC-dex (c) comparison retention of 20 and 70 kDa FITC-dex.

Arterial model fabrication

CAD models of different percentage stenosis were created in SolidWorks, see Figure S3.a&b.1 Afterwards models were 3D printed from 3DM-ABS resin using a Elegoo-Mars 3D printer (Figure S3.a&b.2). Prior to casting the mold were painted with clear lacquer (2X RUST-OLEUM) and left to dry for 24 hours. Transparent smooth plastic slides were glued to the printed mold to form walls and provide smooth surface. A mixture of polydimethylsiloxane (PDMS, Sylgard® 184, silicone elastomer) was poured into the mold, degassed and left to cure overnight. The plastic cover slides were removed, and models were placed in acetone for 24 hours to dissolve the 3D printed mold. After the plastic disintegrated, the mold was taken out of the acetone, and the remnants of the plastic removed. Finally, the resulting model was then placed in an oven at 600C to make sure the acetone was completely evaporated (Figure S3.a&b.3).

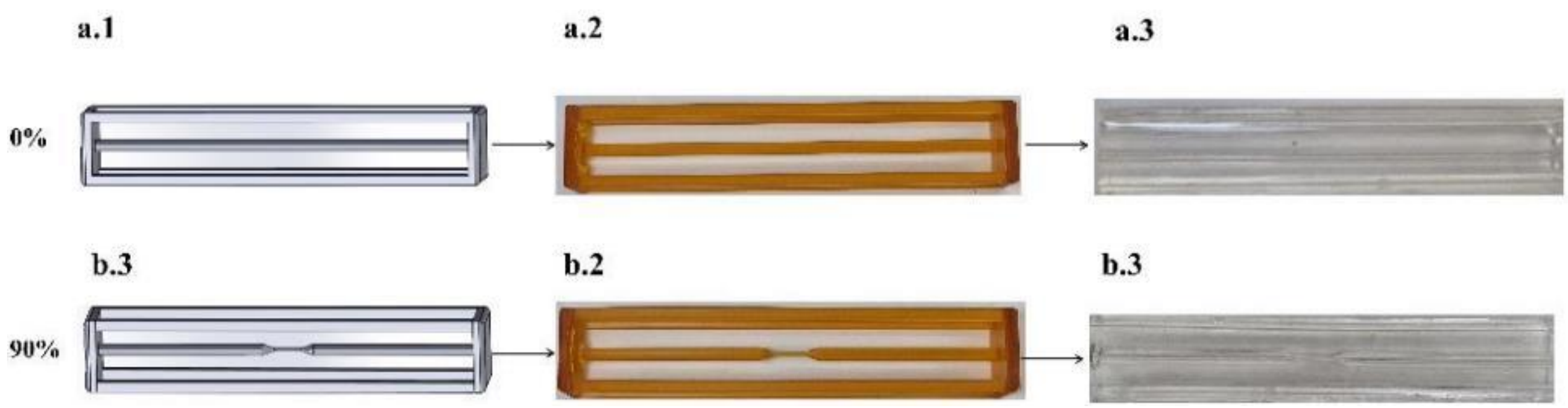

Figure S3. Fabrication of stenosis models (a.1) CAD model (a.2) straight vessel 3D printed model (a.3) Transparent PDMS of straight (0%) model (b.1) 90% stenosis CAD model recapitulating sever stenosis (b.2) 3D printed model of 90% stenosis. (b.3) Transparent PDMS model of 90% stenosis.

Macrophage biological function

Phagocytosis: To evaluate the biological function of CD47 receptor on RBCs and its interaction with macrophages, THP-1 (ATCC) cells were maintained in RPMI medium (Sigma). Cells were cultured at 37°C, in a humidified environment, with 5% CO2. Prior to phagocytosis experiments THP-1 cells were differentiated to macrophage-like-cells using phorbol 12-myristate-13-acetate (PMA, Sigma). THP-1 cells were incubated for 16 hours with PMA at final concentration of 200 ng/ml. After the incubation, freshly media was supplied. Polarized THP-1 cells were incubated with native RBC, sr-RBC and RBC treated with ionomycin to induce eryptosis (e.g., ionomycin) for one hour at 37°C, in a humidified environment, with 5% CO2. Both native RBCs and Ionomycin were labelled with VybrantTM Dil (red) for easier detection. Post incubation cell were gently washed with fresh media to remove suspended cell followed by trypsinization. Phagocytosis was measured using ImageStream®X Mk II (Cytek), image flow cytometry, cells were gated to detect single and focused macrophage cells, and phagocyte event (Supplementary Material and Figure S4a). Phagocytosis mean fluorescent intensity of ionomycin treated cells and native RBCs was compared, as seen in Figure S4b, THP-1 cell incubated with ionomycin treated cells display MFI peak higher than native, above the graph we can see representative macrophage images from this area. The %phagocytosis was calculated by dividing macrophages with fluorescent signal by the total number of macrophages, see Figure S4c.

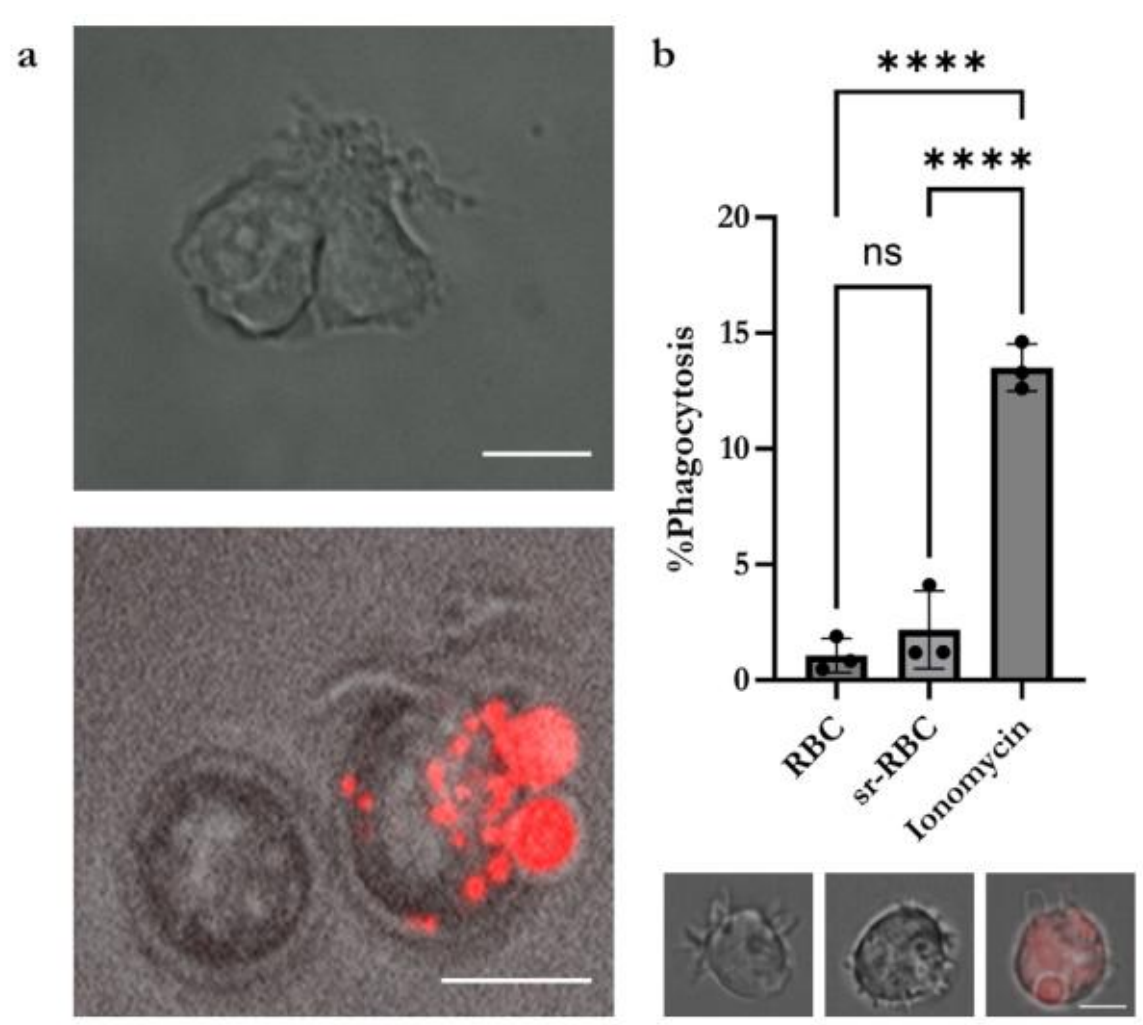

Figure S4. phagocytosis of RBC (a) Polarized THP-1 cells after one hour incubation with sr-RBC (top) and ionomycin treated RBCs (bottom). Positive fluorescent signal was detected after

incubation with ionomycin RBCs suggesting phagocytosis. (b) quantification of %phagocytosis between native RBC, sr-RBC and ionomycin treated RBCs. Significant % phagocytosis is observed in ionomycin whereas for native RBC and sr-RBC lower levels of phagocytosis are present. Statistical significance was determined by one-way ANOVA, ****: p<0.00001, (n = 4 biological replicates). Scale bars 10 µm.

RBC characterization

RBC membrane potential is determined by the protein composition on the membrane, and it is characterized by net negative charge. It may act as an indicator for surface properties and electrostatic stability. Any alteration in the membrane composition, will result in changes in the charge. During sr-RBC fabrication mean corpuscular volume (MCV) decreased, this may be attributed to the loss of hemoglobin and other cytoplasmic proteins during the loading process.

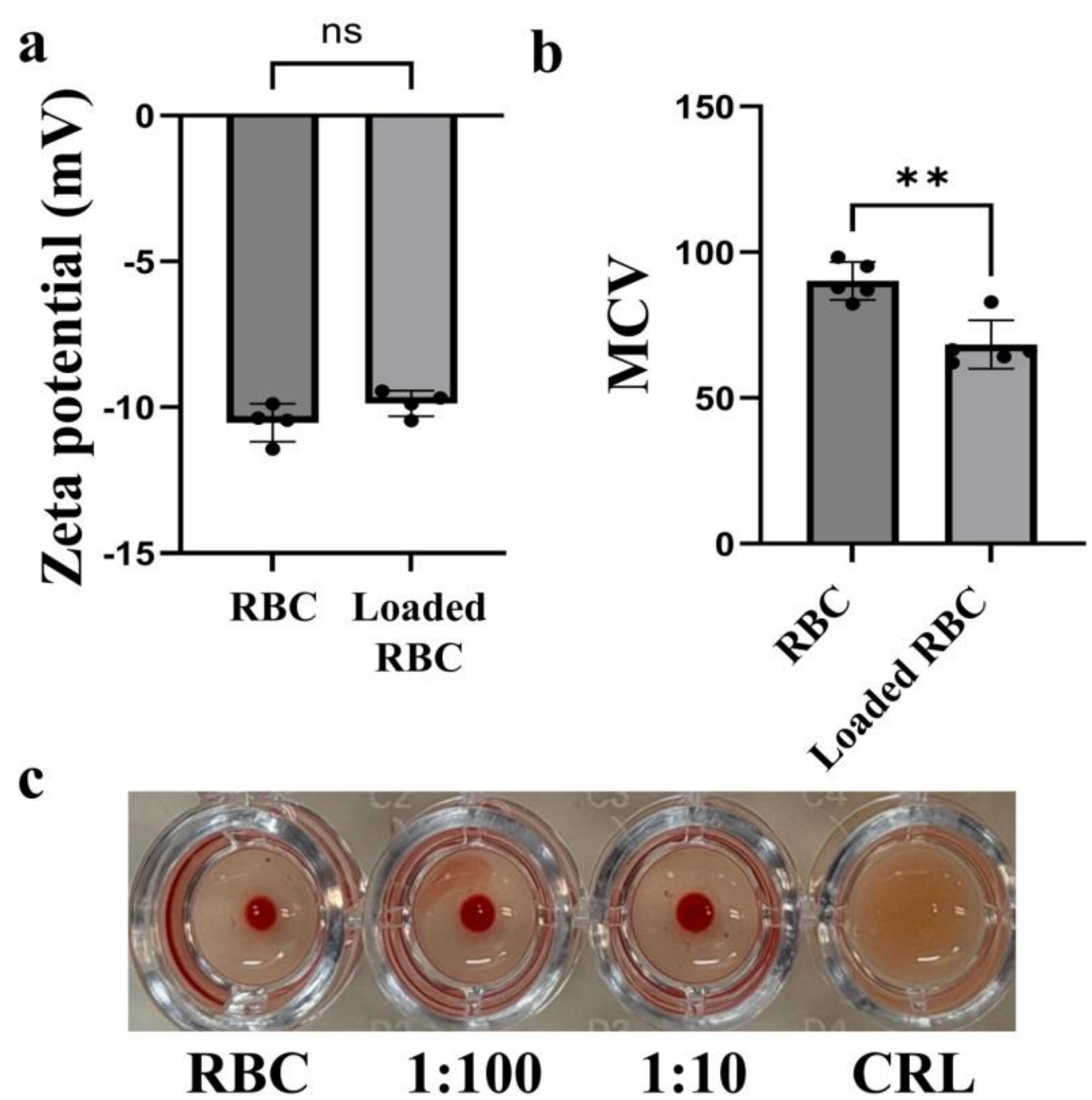

Figure S5. RBC and sr-RBC characterization assays (a) Membrane zeta potential of RBCs and sr-RBCs; no significant difference was observed between the two groups (n = 3 biological replicates from different donors). Statistical significance was assessed using a two-tailed *t*-test;

ns, $p > 0.05$. (b) Mean corpuscular volume of sr-RBCs showed a reduction following the sr-RBC loading process (n = 3 biological replicates from different donors). Statistical significance was assessed using a two-tailed *t*-test; *, $p < 0.05$; **, $p < 0.01$. (c) Representative images (left to right) of RBCs alone, sr-RBCs mixed with RBCs at ratios of 1:100 and 1:10, and a positive control consisting of 500-nm polystyrene nanoparticles mixed with RBCs. sr-RBCs did not induce agglutination, as no visible cell aggregation was observed.

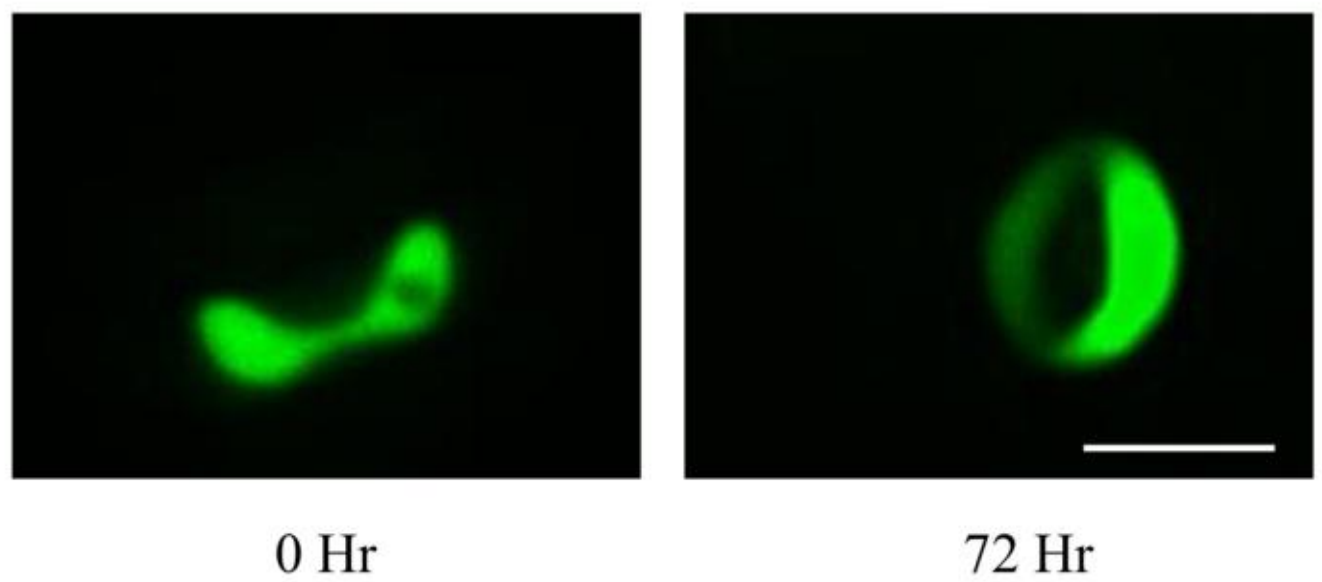

Figure S6. Morphological stability. Confocal imaging of sr-RBC after encapsulation process and after 72 hours of storage. No apparent morphological changed were observed after storage of sr-RBC Scale bar 5 μm.

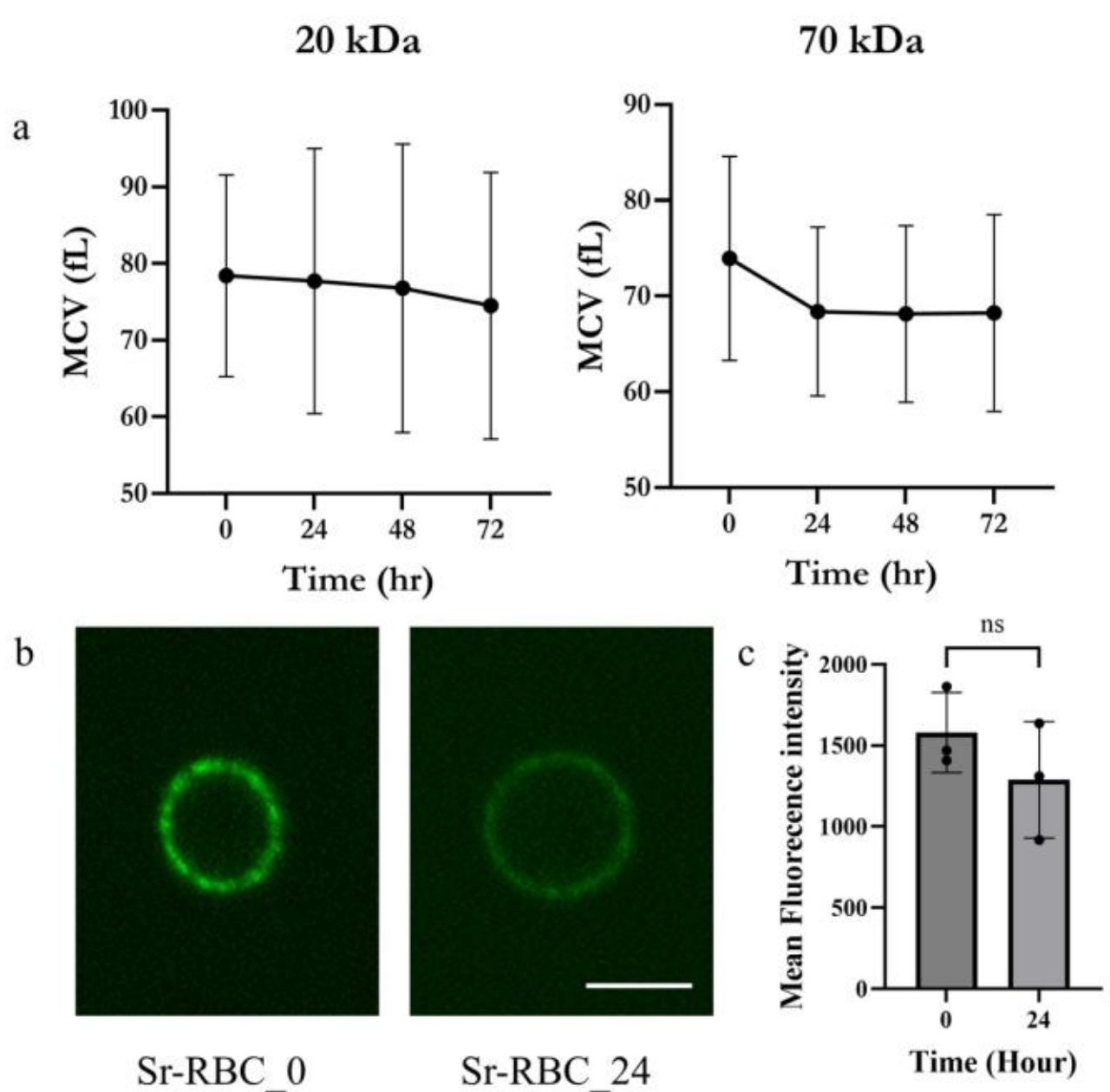

Figure S7. (a) Mean corpuscular volume of sr-RBCs over 72 hours showed a reduction following the sr-RBC loading process (n = 3 biological replicates from different donors). (b) Confocal microscopy of CD47 expression on sr-RBC surface after loading (Sr-RBC_0) and after 24 hours (Sr-RBC_24) storage at 4^0C. scale bar 5µm. (c) Mean fluorescent intensity of CD47 expression at 0- and 24-hour time point. No significant decrease in CD47 expression was observed. Statistical significance was assessed using a two-tailed t-test; ns, $p > 0.05$. (n = 3 biological replicates).

PCN model fabrication

The PCN microfluidic model was developed by Stauber *et al*.[31] The master mold was fabricated using deep reactive ion etching (DRIE) of a silicon wafer, a method well-suited for creating features smaller than 10 µm. The design was created using Clewin software (Figure S8.a.1). A 4-inch, double-side-polished silicon wafer was thermally oxidized to form a 100 nm thick oxide layer. The wafer was then spin-coated with approximately 3 µm of AZ 4533 positive photoresist, baked at 110°C for 2 minutes, and exposed to UV light using an MA6 mask aligner (Karl Suss) at a dose of 43 $mJ \cdot cm^{-2}$. The UV light was projected through a chrome mask containing the model features. After exposure, the wafer was developed in 2.5% TMAH for 120 seconds. The oxide layer in areas not protected by the photoresist was removed using reactive ion etching (RIE) with CHF_3 gas in a Plasma-Therm 790 system. Silicon cavities, 5 µm deep, were then etched using DRIE with a Plasma-Therm Versaline ICP system and a gas mixture of $SF_6/C_4F_8/Ar$. Residual photoresist was removed using commercial solvents (NMP at 75°C and PRS at 75°C), followed by cleaning with Piranha solution (H_2SO_4:H_2O_2). For easier release of PDMS, the master mold was coated with a hydrophobic layer (FTDS) using a molecular vapor deposition (MVD) system. The device was then fabricated from PDMS using soft lithography. After curing overnight at 60°C, the PDMS was peeled from the mold, and 0.5 mm holes were punched to form the inlet and outlet. The device was sealed with a glass slide using oxygen plasma bonding. The master mold can be reused multiple times for fabricating additional PDMS micro-devices. See figure S8.a.2 for microscope image of the final PCN microfluid device.

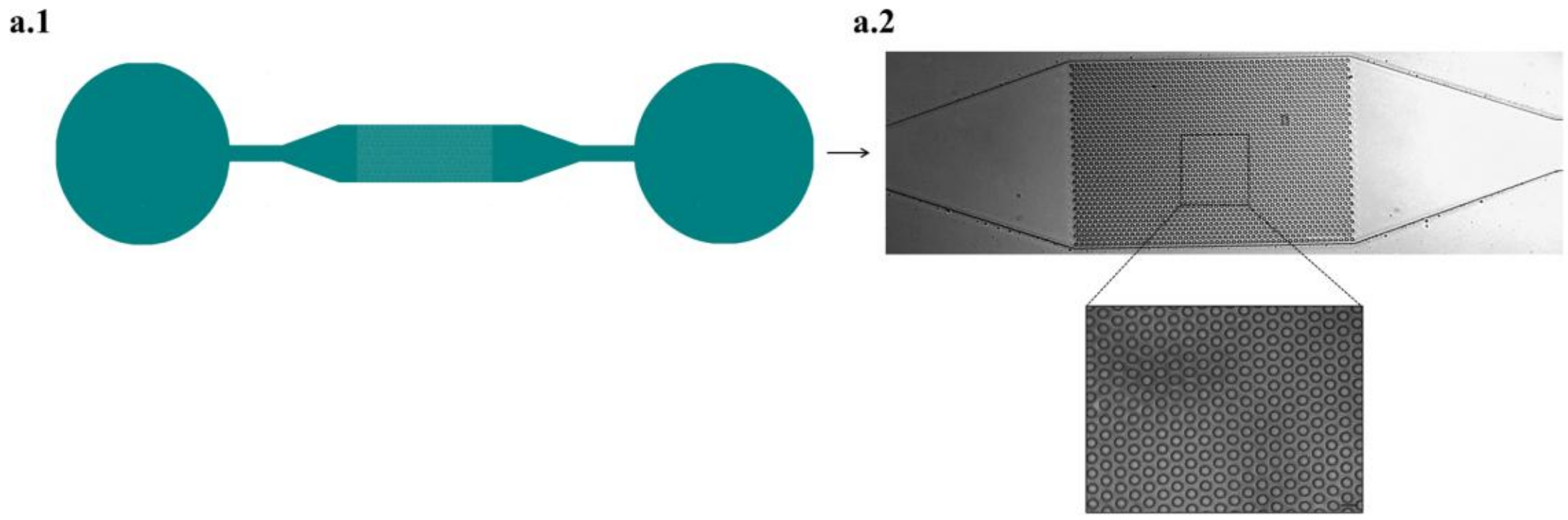

Figure S8. Fabrication of pulmonary capillary network model (a.1) Clewin design of the PCN model features a staggered array of circular pillars, each 5 µm in height and 3.6 µm in diameter, with 5 µm spacing between adjacent pillars. (a.2) Microscope images show the final PCN microfluidic chip made from PDMS after fabrication, scale bar 10 µm. Close-up image of PCN model.

Shear release of 70 kDa FITC-Dex from sr-RBC perfused through 90% stenosis model

Similarly to 20 kDa FITC–dex loading, RBCs were incubated with 70 kDa FITC–dex to evaluate release for a higher-molecular-weight cargo. Significant shear-induced release was observed in the 90% stenosis model, although the release fraction was lower than for 20 kDa FITC–dex, consistent with reduced diffusivity and increased steric constraints for larger cargo.

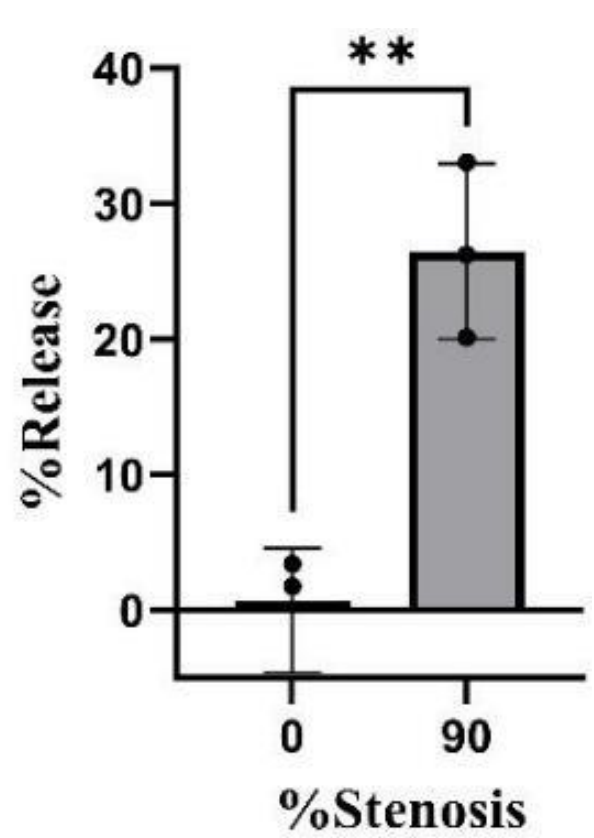

Figure S9. Shear-induced release of 70 kDa FITC-Dextran from RBC carriers. Flow cytometric quantification of released FITC-Dex following transit through straight and 90% stenotic models under identical loading and flow conditions. Data represents mean value of 26.5

±5.3% release in the stenosis case (n=3 biological replicates from different donors). Statistical significance was determined by t-test, *: p<0.05, **: p-value < 0.01.

The Perfusion Systems

For the large stenosis models, a perfusion system was developed by Epshtein *et al*.[40] to replicate arterial flow. The system consists of a fluid reservoir, a peristaltic pump (Watson Marlow C530), and a downstream damper to minimize oscillations from the pump, as shown in Figure S10.a. Approximately 5 µl sr-RBC were mixed with PBS (-/-) for final volume of 300 µl. The solution was perfused in closed circuit though 0, 25, 75 and 90% stenosis models for the duration of one hour. After perfusion samples were taken for further analysis.

The experimental setup for the PCN microfluidic device includes an inverted epi-fluorescence microscope (Ti–Eclipse, Nikon) and an automated pressure control system (Fluigent MFCS-EZ). The device was first washed with PBS to eliminate air bubbles, followed by BSA coating. Then, sr-RBC mixture was perfused through the PCN models at a constant pressure of 4 kPa, see Figure S10.b.

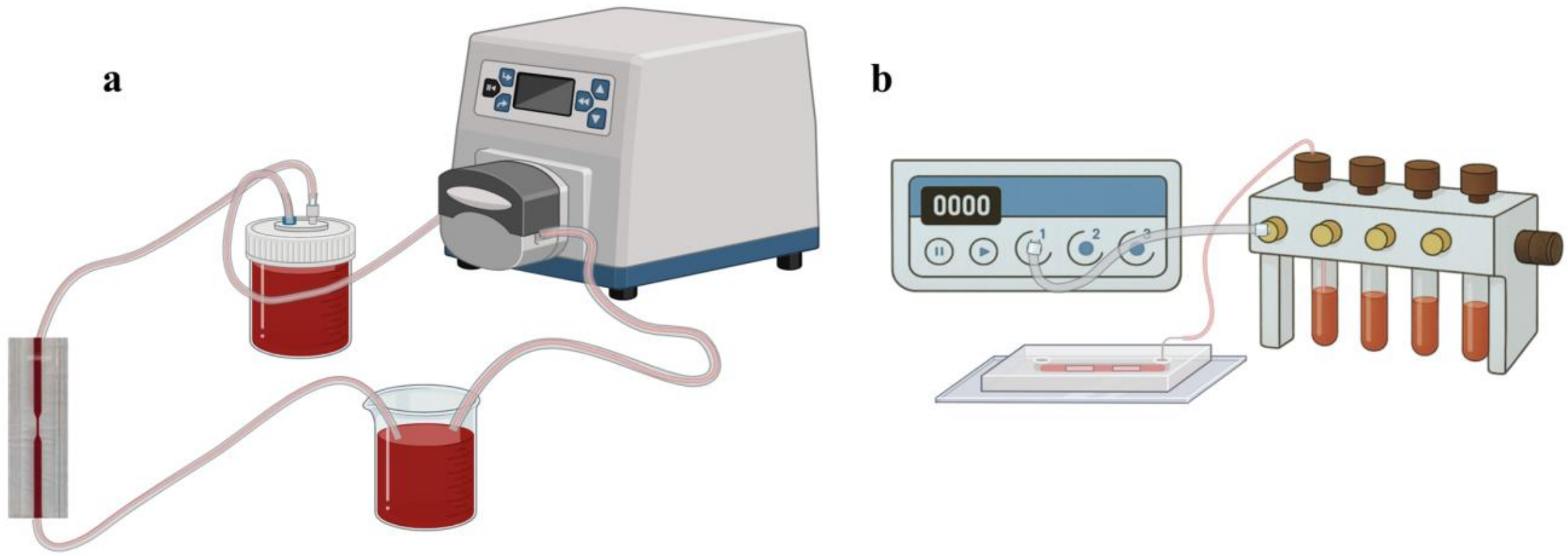

Figure S10. Perfusion system (a) Schematic illustration of the perfusion system. The peristaltic pump draws fluid from a reservoir containing sr-RBC and infuses it into the damper where the air pressure removes the peristaltic oscillations and produces a smooth continuous flow. (b) Schematic

illustration of a Fluigent flow system composed of a microfluidic flow control unit (MFCS™ EZ), which provides constant pressure to perfuse the RBC mixture into the PCN microfluidic model.

Computational Fluid Dynamics - Developed flow along the model

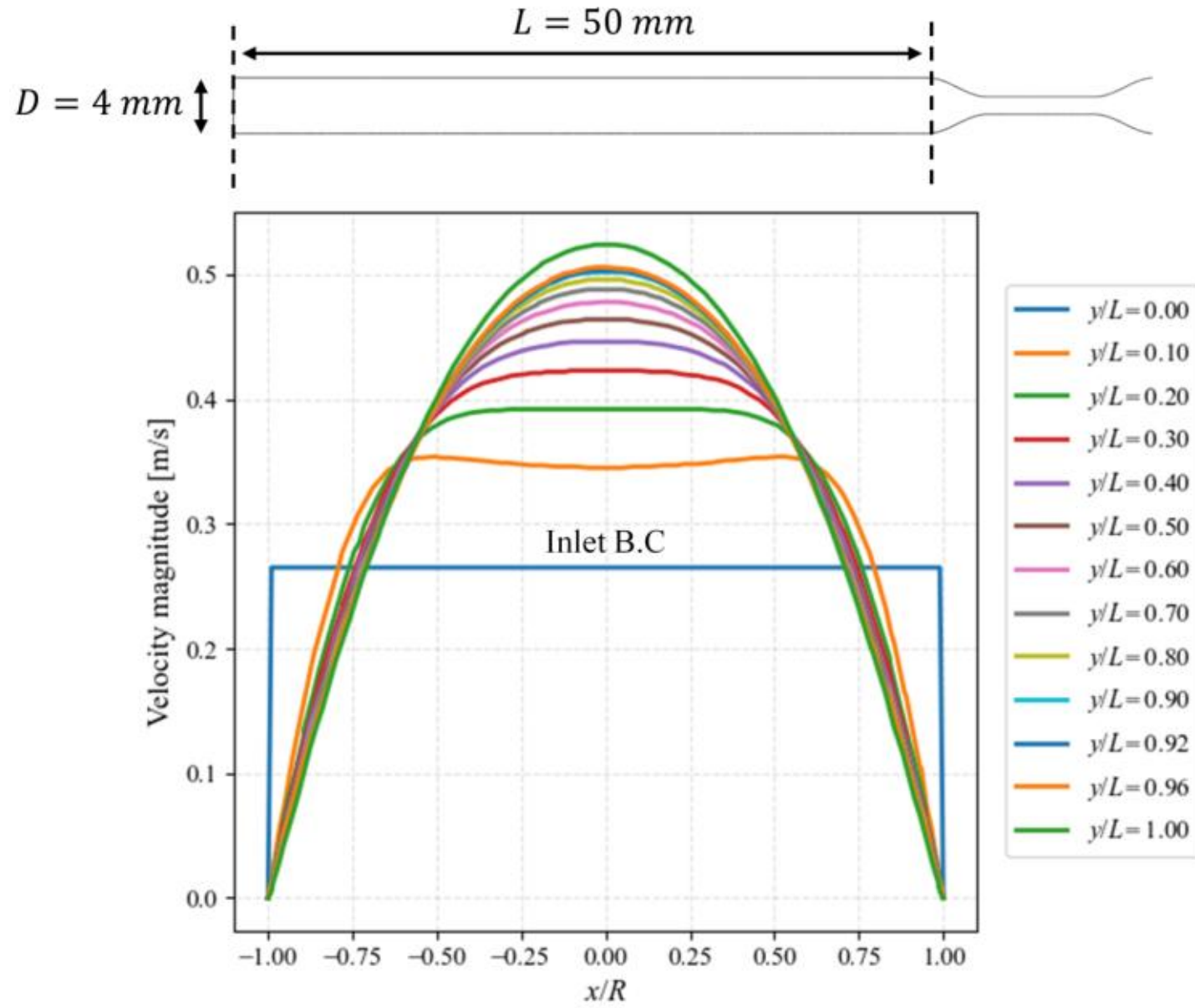

Figure S11. CFD convergences of the flow profile prior to the stenosis region. Velocity magnitude profiles across the model's cross-section, plotted as a function of the normalized radial coordinate x/R ($R = 2\,mm$), at multiple axial locations along the inlet extension. Each curve corresponds to a different normalized axial position y/L ($L = 50\,mm$). The collapse of the profiles indicates that a fully developed velocity distribution is achieved upstream of the stenosis region.

Flow cytometry gating strategies

One million cells were taken for flow cytometry analysis. All flow cytometry experiments began with gating to isolate the singlet, rounded cell population prior to assessing FITC fluorescence. Untreated RBCS were taken as a negative control for unstained cells. Each sample was stopped at 10,000 events, and fluorescent results were quantified as shown in Figures 2f and 3d. Figure S12 shows the representative flow cytometry plots. Additionally, to ensure that reported values reflect intracellular cargo release from intact carriers rather than artifacts, flow cytometry analysis was gated to exclude cellular debris and aggregates, resulting in the exclusion of approximately 2% of the population.

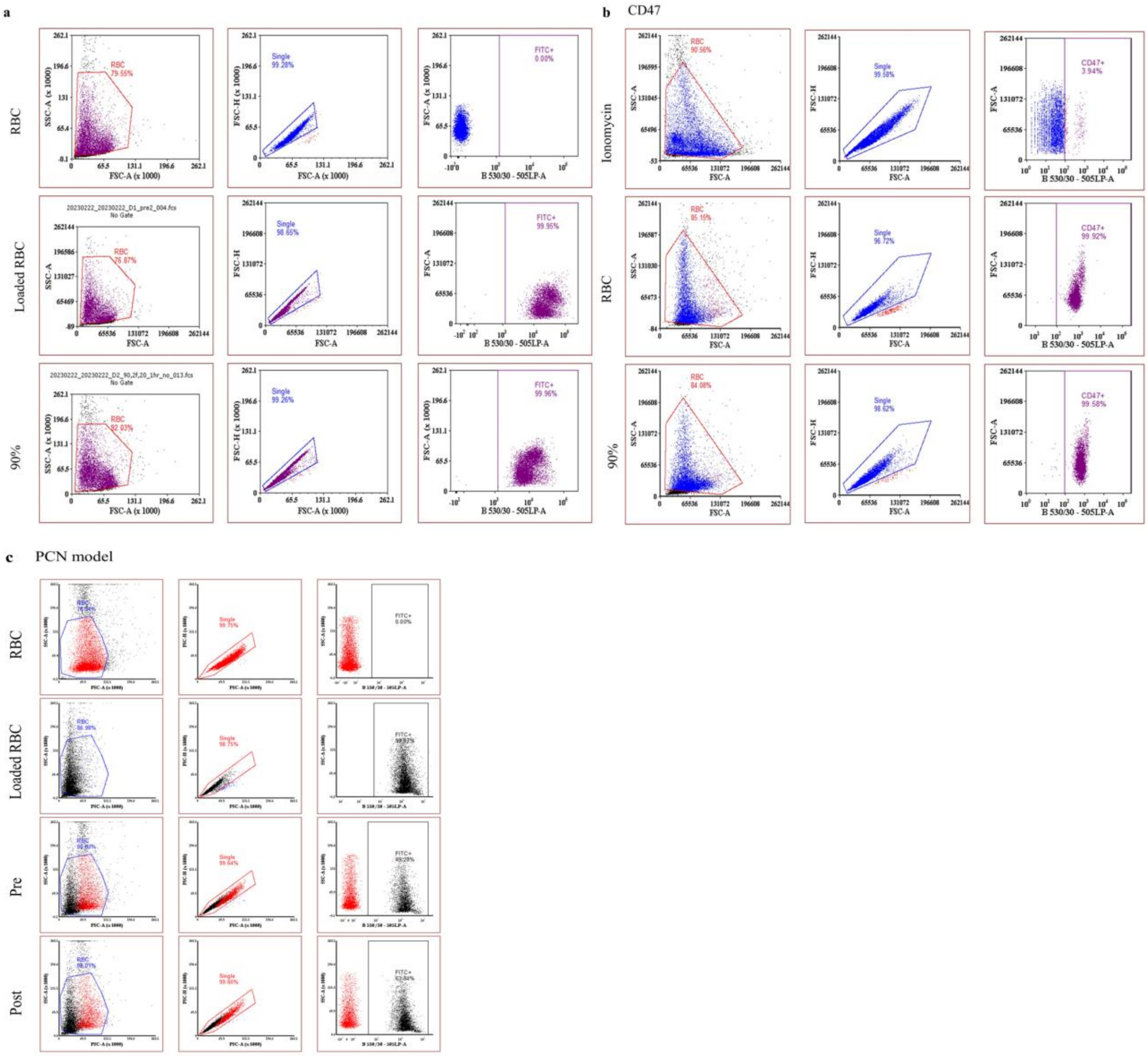

Figure S12. Flow cytometry gating strategies (a) Representative flow cytometry plots showing gating for RBCs. RBC refers to collected erythrocytes with no treatment. Loaded RBC refers to erythrocytes following loading with FITC-dextran; a positive FITC signal was used to define the gate. 90% indicates the population of RBCs after perfusion through the 90% stenosis model. (b) Representative flow cytometry plots showing gating for CD47 expression (c) Representative flow cytometry plots with gating of RBCs in the PCN microfluidic model. RBC prior loading shows untreated erythrocyte, exhibiting no FITC signal. RBC after loading displays a positive FITC signal. Pre and Post denote RBCs sampled before and after perfusion in the PCN microfluidic device, respectively. Loaded RBCs were mixed with unloaded RBCs as described in the Methods.

Table S1.

Properties used in RBC simulation

**Plasma Properties**	
Density, r, (g/cm3)	1
Viscosity, m, (Pa s)	0.0012
**Cell Membrane Properties**	
Viscosity ratio of RBC cytoplasm to plasma	5
Shear modulus, G (dyne/cm)	0.0063
Membrane viscosity (Pa s)	0.128
Elastic area compression modulus, K (dyne/cm)	0.046
Bending modulus, J	2.4e-19
Volume, mm^3	94
Area, mm^2	135

Legends for Movies

Video 1: Time-lapsed microscopy imaging of sr-RBCs (green) mixed with RBCs at 30% hematocrit, perfused through the PCN microfluidic device under a working pressure of 4 kPa, corresponding to the physiological velocities of pulmonary capillaries, showing the sr-RBCs do not get entrapped in the PCN.

Video 2: Suspension dynamics and the areal strain distribution of a single RBC traversing a 25% stenosis, quantified from the cellular blood flow simulations. The inset shows the time-varying shear rate driving the RBC dynamics through the stenosis. The RBC undergoes tank-treading motion as it translate through the stenosis. The moving cursor indicates the current shear rate exerted on the RBC. The shear is imposed along the x-direction.

Video 3: Suspension dynamics and the areal strain distribution of a single RBC traversing a 75% stenosis, quantified from the cellular blood flow simulations. The inset shows the time-varying shear rate driving the RBC dynamics through the stenosis. The RBC undergoes tank-treading motion as it translate through the stenosis. The moving cursor indicates the current shear rate exerted on the RBC. The shear is imposed along the x-direction.

Video 4: Suspension dynamics and the areal strain distribution of a single RBC traversing a 90% stenosis, quantified from the cellular blood flow simulations. The inset shows the time-varying shear rate driving the RBC dynamics through the stenosis. The RBC undergoes tank-treading motion as it translate through the stenosis. The moving cursor indicates the current shear rate exerted on the RBC. The shear is imposed along the x-direction.

Video 5: Time lapse microscopy video of showing THP-1 cells incubated with sr-RBCs (left, in green) and ionomycin treated RBC (right, in orange)**.** Images were taken every 10 s during 1hour incubation. Scale bar: 10 μm.
